# Prevalence and incidence of orthostatic hypotension in patients with Parkinson’s disease: an updated systematic review and meta-analysis

**DOI:** 10.3389/fneur.2026.1751756

**Published:** 2026-02-12

**Authors:** Lijuan Wang, Xingxing Su, Ping Zhu, Jing Wang

**Affiliations:** 1 Department of Neurosurgery, Tangdu Hospital, Fourth Military Medical University, Xi'an, China; 2 Department of Hematology, Tangdu Hospital, Fourth Military Medical University, Xi'an, China

**Keywords:** Parkinson’s disease, orthostatic hypotension, prevalence, incidence, systematic review, meta-analysis

## Abstract

**Aim:**

Systematically quantifying the prevalence and incidence of orthostatic hypotension in Parkinson’s disease patients.

**Background:**

Orthostatic hypotension is a common non-motor symptom in Parkinson’s disease patients. Although systematic reviews have been published previously, existing data are outdated, and there is a lack of research examining differences in the prevalence and incidence of this symptom across various etiologies. An update of current studies is urgently needed.

**Design:**

Systematic review and meta-analysis.

**Methods:**

A systematic search was conducted across the following databases: Medline, PubMed, Web of Science, Embase, Cochrane Library, CINAHL, and ProQuest, retrieving English-language publications since each database’s inception. Included studies comprised cross-sectional, retrospective cohort, and prospective cohort investigations reporting the prevalence or incidence of orthostatic hypotension in patients with Parkinson’s disease.

**Results:**

This meta-analysis included 55 studies involving 10,463 patients with Parkinson’s disease. The combined prevalence of orthostatic hypotension (OH) was 33% (95%*CI*, 28–37%), with high heterogeneity among studies (*I*^2^ = 96%). Subgroup analysis revealed a significantly higher prevalence in males (24%) compared to females (12%). Regionally, Europe reported the highest prevalence (42%), followed by Asia (32%) and North America (28%). Prevalence rates obtained using active standing blood pressure measurement (33%) were slightly higher than those measured via tilt table testing (28%). The combined prevalence of neurogenic orthostatic hypotension was 26%. The prevalence of symptomatic and asymptomatic orthostatic hypotension was similar (20 and 22%, respectively). Meta-regression analysis indicated that mean patient age was the primary factor explaining heterogeneity between studies (adjusted *τ*^2^ reduced by 94.9%), while disease duration, MDS-UPDRS- III score, and mean levodopa equivalent daily dose showed no significant association with orthostatic hypotension prevalence. However, it must be emphasized that these findings may be constrained by significant heterogeneity among studies, inconsistencies in variable definitions and measurement methods across included studies, and the limited number of available data points for regression analysis. Publication bias analysis suggested a small-sample effect; after adjustment using trimmed and inflated methods, the estimated prevalence decreased to 24.9%.

**Conclusion:**

Orthostatic hypotension is prevalent in Parkinson’s disease with a combined prevalence of 33%, exhibiting considerable heterogeneity. This heterogeneity stems from variations in clinical practice and research methodologies. Our meta-analysis revealed that, at the pooled level, the prevalence of orthostatic hypotension showed no significant association with disease duration, MDS-UPDRS-III score and mean levodopa equivalent daily dose. However, this likely reflects inconsistencies in case definitions, timing of measurements, and measurement settings. Future studies must employ standardized autonomic assessment methods to accurately define neurogenic orthostatic hypotension and identify reliable clinical correlates.

**Systematic review registration:**

https://www.crd.york.ac.uk/PROSPERO/view/CRD42025632838, identifier PROSPERO (CRD42025632838).

## Highlights

- The management of orthostatic hypotension requires the involvement of clinical nursing staff.
- Parkinson’s disease patients have an increased risk of orthostatic hypotension as the disease progresses.
- Falls caused by orthostatic hypotension can be prevented through preventive interventions.
- The updated meta-analysis indicates a pooled prevalence of orthostatic hypotension in Parkinson's disease patients of 33% (or 24.9% when adjusted for small-study effects), with a neurogenic subtype accounting for 26%. The prevalence of asymptomatic OH is substantial (22%), highlighting the critical need for systematic screening beyond symptom inquiry.
- Regional prevalence varies significantly, with the highest estimate in Europe (42%), followed by Asia (32%). Currently, there is a notable lack of data from Africa and Oceania, indicating an important gap in global epidemiological understanding.
- The quality of evidence remains heterogeneous. Future primary studies require more transparent and standardized reporting on potential influencing factors, such as disease stage, comorbidities, concurrent medications, and assessment timing to facilitate more precise risk factor exploration in subsequent evidence synthesis.

## 1 Introduction

Parkinson’s disease (PD) is a degenerative disorder of the central nervous system that primarily affects middle-aged and older adults. It is projected that by 2050, the global prevalence of Parkinson’s disease will reach 25.2 million cases, representing an increase of 112% compared to 2021 (1). Orthostatic hypotension (OH) is a common non-motor symptom of Parkinson’s disease, resulting from autonomic dysfunction. It manifests as symptoms of orthostatic intolerance, such as dizziness, blurred vision, weakness, and orthostatic syncope, when patients change their body position (2), leading to falls (3) and other adverse events. Patients may even become sedentary due to fear of falling, severely impacting their safety, quality of life, and social participation. Additionally, studies have shown that OH increases the risk of dementia and cognitive impairment in Parkinson’s disease patients (4).

However, existing literature reports significant heterogeneity in the prevalence and incidence of OH among PD patients. This discrepancy stems not only from differences in study populations and diagnostic methods but is also influenced by a fundamental methodological limitation: most studies fail to distinguish between the etiologies of OH, namely neurogenic orthostatic hypotension (nOH) and secondary orthostatic hypotension. Neurogenic orthostatic hypotension arises from autonomic nervous system degeneration in Parkinson’s disease patients, leading to vascular constriction failure upon standing. In contrast, secondary orthostatic hypotension is primarily attributable to exogenous factors, particularly dopaminergic medications and other drugs used to treat comorbidities such as hypertension. This lack of differentiation may significantly overestimate the true epidemiological burden of autonomic dysfunction associated with the inherent pathology of Parkinson’s disease.

Although scholars have conducted a systematic review of the prevalence of orthostatic hypotension in Parkinson’s disease patients, their analysis only searched the Medline and Embase electronic databases, and included the results of 25 studies published before December 2009 that met the criteria. The analysis showed that the prevalence of orthostatic hypotension in Parkinson’s disease patients was 30.1% (5). However, the available evidence concerning the epidemiological distribution of OH etiologies within the Parkinson’s disease population remains sparse. Moreover, the influence of diagnostic heterogeneity on pooled prevalence estimates has not been systematically evaluated. Therefore, further understanding of OH incidence and prevalence may help improve clinical management of OH. In summary, we conducted a systematic review and meta-analysis to summarize the existing global epidemiological data on the incidence of orthostatic hypotension in Parkinson’s disease patients.

## 2 Methods

This study was prospectively registered in the International Prospective Register of Systematic Reviews (PROSPERO: 2025 CRD42025632838).

### 2.1 Design

This systematic review and meta-analysis was reported using the Preferred Reporting Items for Systematic Reviews and Meta-Analyses (PRISMA) guidelines (6) and a 24-step guide on how to design, conduct, and successfully publish a systematic review and meta-analysis in medical research (7).

### 2.2 Search strategy

We conducted a comprehensive literature search across multiple databases, including Medline (via EBSCOhost), PubMed, Web of Science, Embase, the Cochrane Library, CINAHL, ProQuest, covering the inception of each database up until 28th Dec, 2024. Additionally, manual searches of reference lists were performed to ensure the comprehensiveness of our study selection process. Our search approach combined both free terms and subject terms, utilizing Boolean operators OR and AND. The search methodologies adopted were as follows: (“Parkinson’s disease” OR “PD” OR “spasmus agitans” OR “parkinsons” OR “parkinsonism”) AND (“orthostatic hypotension” OR “postural hypotension” OR “hypotension”) AND (“prevalence” OR “epidemiology” OR “incidence” OR “morbidity” OR “rate”) and the details were shown in Table 1.

**Table 1:** Inclusion and exclusion criteria for the systematic review.

Domain	Inclusion	Exclusion
Participants	Patients with a confirmed diagnosis of Parkinson’s disease	Population heterogeneity
Study setting	Hospitalization department or Outpatient department	
Study design	Observational studies/cross-sectional studies/prospective cohort studies/retrospective cohort studies.	Qualitative studies/experimental Studies (randomized control or Nonrandomised Control)/case studies/reviews/Conference papers
Study outcome	Prevalence/incidence/number of cases of orthostatic hypotension/orthostatic hypotension prevalence versus overall sample.	Postural and non-postural hypotension as baseline
Type of publication	Peer reviewed full text papers	
Study period and language		Non-English language articles and studies

### 2.3 Study selection

All retrieved study inscription were imported into NoteExpress software (version 3.7.0) for duplicate removal. Two independent reviewers conducted primary screening by assessing titles and abstracts against the predetermined eligibility criteria. Full-text articles were retrieved for eligibility confirmation through comprehensive review if at least one reviewer identified potential relevance. The operationalized inclusion/exclusion criteria with are detailed in Appendix 1.

### 2.4 Data extraction

Two researchers independently extracted data from the literature, followed by dual cross-verification. Extracted information included: authors, title, publication year, study region, study design, study population (age, gender, disease duration, disease stage), total sample size, number of orthostatic hypotension (OH) cases, diagnostic criteria for Parkinson’s disease, definition of orthostatic hypotension, OH diagnostic methods, follow-up duration (number of OH cases at different time points), and use of medications potentially affecting outcomes (e.g., antihypertensives).

### 2.5 Risk of bias assessment

This study selected a risk bias assessment tool specifically developed for epidemiological research (8). The tool consists of 10 items, designed to assess external validity (items 1–4) and internal validity (items 5–10). Quality evaluation was primarily conducted from four aspects: selection bias, non-response bias, measurement bias, and analysis bias. The total score ranges from 0 to 10 points. Each item is answered as ‘Yes’ (low risk, scored as 1 point), “No” (high risk, scored as 0 points), or ‘Uncertain’ (reason must be explained, typically scored as 0 points). A total score of 0–5 indicates high risk, 6–7 indicates moderate risk, and 8–10 indicates low risk. Two researchers independently conducted the quality assessment for each study, cross-checked the assessment results, and resolved any discrepancies through a third-party discussion in a group meeting.

### 2.6 Data synthesis

This study conducted statistical analyses using the metaprop command in Stata 15.0. Pooled estimates were derived by applying an inverse variance model combined with a Freeman–Tukey double arcsine transformation. Heterogeneity across studies was assessed using Cochrane’s *Q* test and the *I*^2^ statistic. A *p* < 0.05 in Cochrane’s *Q* test was considered indicative of significant heterogeneity (9). The *I*^2^ statistic describes the proportion of total variation across studies that is due to heterogeneity rather than chance, with values <25%, 25–75, and >75% representing low, moderate, and high heterogeneity, respectively. Given that high heterogeneity is common in incidence and prevalence studies (10), we anticipated that variability in reported rates could be explained by factors such as patient demographics (sex, age), disease progression and stage, diagnostic criteria and methods for orthostatic hypotension, and underlying etiology. Therefore, subgroup analyses were performed accordingly. For continuous variables (e.g., age, disease duration), meta-regression was employed; a *p*-value < 0.10 was regarded as statistically significant in these analyses due to the typically low statistical power of such tests (11, 12). Publication bias was evaluated visually using funnel plots and statistically tested with Egger’s linear regression method, where *p* < 0.10 suggested asymmetry. When asymmetry was detected, a trim-and-fill procedure was applied to adjust for potential publication bias.

## 3 Results

### 3.1 Selection process

**Figure 1:**
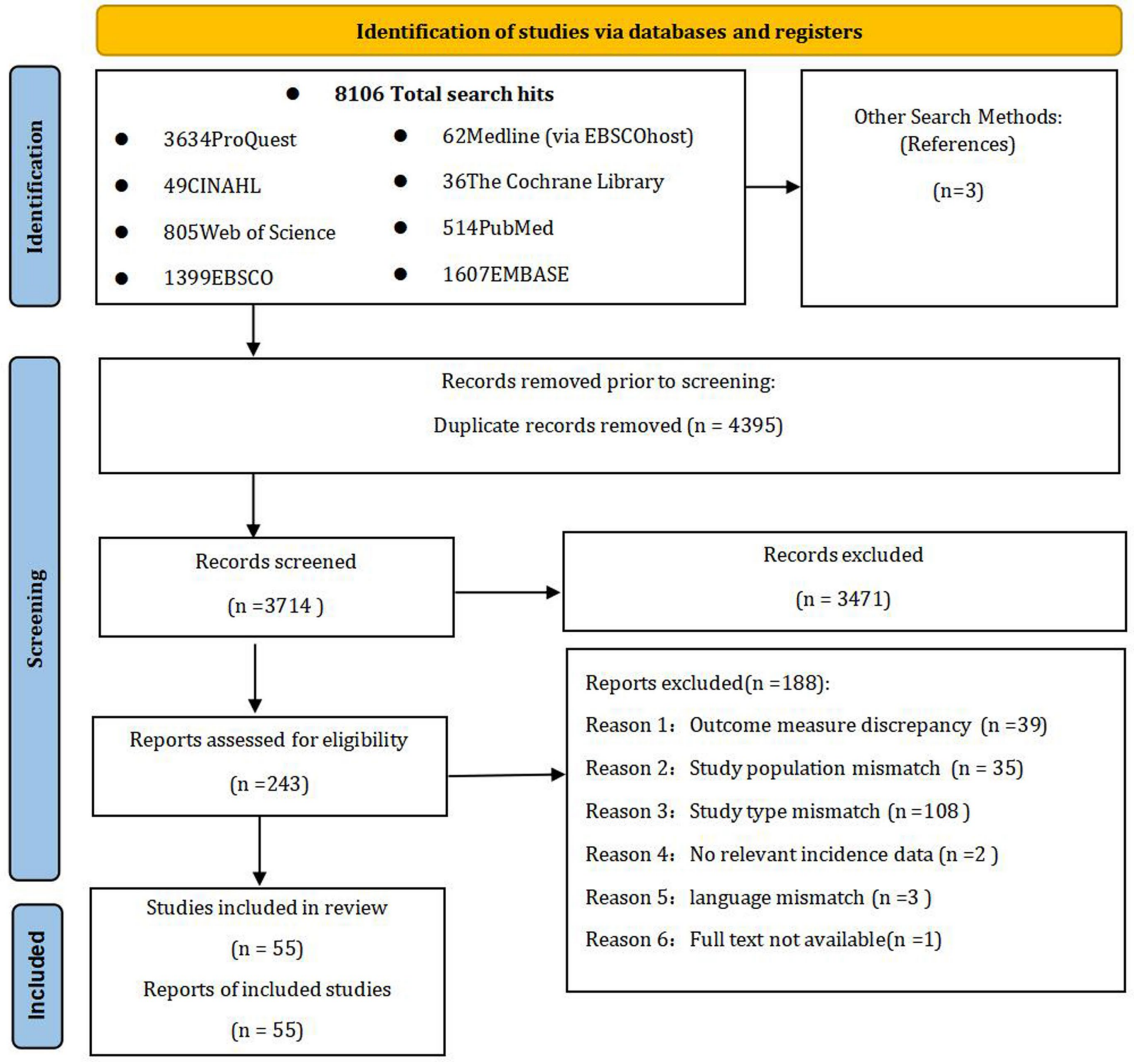
Flowchart of study selection. This diagram was adapted from the template provided by PRISMA under the terms of the Creative Commons Attribution License (CC BY) (87).

Through systematic database searches, we identified 8,106 records. Manual searches of reference lists identified an additional three records. Following duplicate removal, 3,714 unique records were screened based on titles and abstracts. This led to the exclusion of 3,471 records, primarily due to irrelevant patient populations, a focus other than orthostatic hypotension, or a non-epidemiological study design. Full-text articles were retrieved and assessed for the remaining 243 studies, of which 188 were excluded. The main reasons for exclusion at this stage were ineligible study populations and ineligible outcome measures or data. Finally, 55 studies fulfilled all inclusion criteria and were included in the systematic review and meta-analysis. These comprised 39 cross-sectional studies, 11prospective cohort studies, and five retrospective cohort studies. The study selection process is detailed in the PRISMA flow diagram (Figure 1).

### 3.2 Study characteristics

This study included 55 research studies involving a total of 10,463 participants. The sample size for Parkinson’s disease patients ranged from 48 to 1,227 individuals, encompassing 18 multicenter studies. These studies collectively involved 2,793 Parkinson’s disease patients with orthostatic hypotension from 12 countries. Among the included studies, 18 were conducted in Europe, 4 in North America, 24 in Asia, and 9 were cross-regional studies. In the 54 studies reporting gender distribution, 4,081 participants were female and 5,878 were male. Extracted study characteristics included first author, publication year, region, sample size, study design, and methods for diagnosing orthostatic hypotension (Table 2).

**Table 2:** Summary of details and findings of included studies (*n* = 55).

First author(s) (year)	Study location	Study design	Male/Female	Age (years), mean (SD) or as age groups or range (as reported by authors)	Disease duration (years/month) mean (SD) /median (IQR)/as age groups or range	Levodopa equivalent dose (mg/day) mean (SD)/median (IQR)	OH measuring instruments	Prevalence/Incidence (case/N)
(34)	Europe	Prospective Cohort Study	34/16	65.06 ± 9.15	8.23 ± 5.13	736 ± 386	A	17/50
(35)	Europe	Cross-sectional Study	109/66	PD-OH:72.4 ± 7.5 PD-NOH:69.2 ± 8.4	PD-OH: 3.5 (8.0) PD-NOH: 5.0 (8.7)	PD-OH: 400 (350) PD-NOH: 300 (425)	A	87/175
(36)	Multiple regions	Cross-sectional Study	70/51	PD-OH: 67.77 ± 6.20 PD-NOH: 65.75 ± 10.12	NA	Symptomatic OH(+) 56.4 ± 74.0 Asymptomatic OH(+) 52.6 ± 85.5	A	37/121
(37)	North America	Cross-sectional Study	209/108	71.0 ± 10.2	11.6 ± 5.6	PD + OH 1,153.7 ± 656.4 PD without OH 1,160.9 ± 860.4	A	93/317
(38)	Multiple regions	Prospective Cohort Study	79/43	65.87 ± 8.83	10.34 ± 5.98	NA	A	38/122
(39)	Multiple regions	Prospective Cohort Study	NA	29–85	<2	NA	A	35/907
(40)	Asia	Cross-sectional Study	61/28	59.85 ± 10.54	1–5	NA	A	21/89
(41)	Multiple regions	Prospective Cohort Study	385/275	63 (55, 70)	2 (1, 4)	0 (0, 405.25)	A	88/660
(42)	Asia	Cross-sectional Study	58/43	66.6 ± 8.2	3 (1, 4)	277.0 ± 238.7	A	26/101
(43)	Asia	Cross-sectional Study	108/42	50–80	NA	PD-OH 0–750 mg: *n* = 52 >751 mg: *n* = 6	A	62/150
(44)	Multiple regions	Prospective Cohort Study	264/139	64.2 (9.7)	7.1 (7.3)	NA	A	54/403
(45)	Europe	Cross-sectional Study	102/83	64.6 ± 9.7	≤1	355.0 ± 434.0	A	52/185
(46)	North America	Prospective Cohort Study	47/17	PD-OH: 72 ± 1 PD-NOH: 60 ± 2	NA	NA	A	25/62
(47)	North America	Cross-sectional Study	756/369	71.1 ± 10.3	135.2 (81.9)	PD-OH: 847.6 ± 479.8 PD-NOH: 878.8 ± 547.2	A	198/1,125
(48)	Europe	Cross-sectional Study	36/28	78.8 ± 6.5	9.9 (6.8)	NA	A	33/64
(20)	Asia	Cross-sectional Study	101/87	68.4 ± 10.49	3.4 ± 3.7	NA	B	56/188
(49)	Asia	Cross-sectional Study	47/53	65.5 ± 11.6	9.2 ± 6.1	NA	A	18/100
(50)	Europe	Cross-sectional Study	NA	PD-OH:72.6 ± 8.1 PD-NOH:68 ± 9.6	NA	NA	A	42/89
(51)	Asia	Cross-sectional Study	186/132	66.1 ± 9.5	PD-OH: 8.0 (6.0) PD-NOH: 6.0 (7.0)	564.6 ± 394.1	A	114/318
(52)	Asia	Cross-sectional Study	78/72	PD-OH: 70.24 ± 8.06 PD-NOH: 62.07 ± 10.49	PD-OH 5.00 (3.00–8.00) PD-NOH 3.50 (2.00–6.00)	NA	A	49/150
(53)	North America	Cross-sectional Study	149/77	PD-OH: 71.0 ± 9.3 PD-NOH: 64.8 ± 10.8	≥3.5	NA	A	69/226
(54)	Europe	Cross-sectional Study	80/40	68.2 ± 10.1	5.8 ± 4.7	NA	A	63/120
(55)	Asia	Cross-sectional Study	102/69	PD-OH: 65.28 ± 11.44 PD-NOH: 65.23 ± 10.58	PD-OH: 8.0 (6.0, 11.0) PD-NOH: 6.0 (4.0, 10.0)	PD-OH: 750.0 (600.0, 929.7) PD-NOH: 475.0 (307.5, 650.0)	NA	80/171
(56)	Europe	Cross-sectional Study	79/35	64 ± 10	6.0 ± 4.0	698.7 ± 366.9	A	51/114
(57)	Multiple regions	Cross-sectional Study	121/89	66 ± 11	NA	NA	A	105/210
(58)	Europe	Cross-sectional Study	26/22	PD-OH: 64.96 ± 9.7 PD-NOH: 65.6 ± 8.7	≥5	PD-OH: 1,036.87 ± 387.4 PD-NOH: 864.2 ± 446.1	B	23/48
(59)	Asia	Prospective Cohort Study	NA	PD-OH: 66 (12) PD-NOH: 60 (8)	PD-OH: 90 (84) PD-NOH: 72 (63)	PD-OH: 547 (446.7) PD-NOH: 500 (296.8)	A	17/80
(60)	Multiple regions	Cross-sectional Study	131/86	59.34 ± 9.80	≤2	NA	A/D	24/217
(61)	Europe	Retrospective Cohort Study	80/52	61.4 ± 11.9	16.4 ± 8.8	NA	A	67/132
(62)	Asia	Cross-sectional Study	74/63	64.1 ± 10.5	10 ± 6.2	957.2 ± 360.1	A	69/137
(63)	Asia	Cross-sectional Study	54/44	69.4 ± 9.4	≥5	NA	B	33/98
(64)	Europe	Cross-sectional Study	73/30	66.5 ± 0.9	9.4 ± 0.6	LDED > 1,050 mg/day PD-NOH: *n* = 31 PD-NOH: *n* = 19	A	38/103
(65)	Europe	Cross-sectional Study	43/48	66 ± 9	6–12	NA	A	53/91
(66)	Asia	Cross-sectional Study	83/49	PD-NOH: 68 (39–90)	≤3	NA	B	74/132
(67)	Asia	Cross-sectional Study	57/25	69.2 + 10.3	PD-OH: 6.0, (3, 9.5) PD-NOH: 3.0, (1, 8)	NA	A	33/82
(68)	Asia	Cross-sectional Study	116/92	61.7 ± 7.0	4.4 ± 3.1	NA	B	35/208
(69)	Asia	Retrospective Cohort Study	49/71	62.4 ± 10.3	11.2 ± 6.7	970.5 ± 402.5	A	59/120
(70)	Asia	Cross-sectional Study	61/36	67 ± 10	7.0 ± 6.0	NA	E	51/97
(71)	Asia	Cross-sectional Study	75/64	≥40	5.1 ± 4.7	NA	B	25/139
(72)	Europe	Cross-sectional Study	88/34	65 ± 9	6.3 ± 3.9	671 ± 372	A	53/122
(73)	Europe	Cross-sectional Study	81/32	64.8 ± 10.2	6.5 ± 4.1	693 ± 371	A/C	53/113
(74)	Asia	Cross-sectional Study	105/53	PD-OH: 67.18 ± 7.95 PD-NOH: 63.20 ± 12.07	PD-OH: 7.41 ± 4.21 PD-NOH: 6.27 ± 3.98	NA	A	66/158
(75)	Multiple regions	Retrospective Cohort Study	202/95	62.1 (54.8–68.55)	≤3	NA	A	42/297
(76)	Asia	Cross-sectional Study	55/35	58.8 ± 10.8	NA	NA	A/C	16/90
(77)	Asia	Cross-sectional Study	67/49	PD-OH: 66.00 (61.00, 72.00) PD-NOH: 67.00 (58.50, 73.00)	PD-OH: 4.00 (2.00, 7.00) PD-NOH: 3.00 (1.00, 6.00)	NA	A	27/116
(78)	Asia	Cross-sectional Study	37/36	70.8 ± 8.7	PD-OH: 1.0 (1.3) PD-NOH: 1.0 (1.0)	NA	B	20/73
(79)	Asia	Cross-sectional Study	66/58	70.1 ± 8.9	12.0 (18.0)	NA	B	34/124
(80)	Asia	Retrospective Cohort Study	63/70	67.2 ± 9.9	9.3 (6.6)	756 ± 423.0	A	148/1,227
(81)	Asia	Cross-sectional Study	100/88	61.2 ± 11.7	4.3 ± 3.8	418.6 ± 332.8	A	81/188
(82)	Europe	Prospective Cohort Study	27/35	73.5 ± 9.9	NA	NA	A	25/62
(18)	Europe	Cross-sectional Study	27/31	70.9 ± 9.7	5.1 ± 6.4	1,128.3 ± 648.9	A	28/58
(83)	Europe	Prospective Cohort Study	44/39	69.2 ± 10.0	6.6 ± 0.8	NA	A/B	20/83
(84)	Multiple regions	Prospective Cohort Study	30/20	64.3 ± 9.4	12.8 ± 6.1	935.9 ± 538.8	A	13/50
(85)	Europe	Retrospective Cohort Study	73/26	64.0 ± 10.1	6.4 ± 4.0	662.0 ± 352.7	A	46/99
(86)	Europe	Prospective Cohort Study	67/38	61 ± 9	<3	NA	B/D	7/105

Parkinson’s disease (PD); Orthostatic hypotension (OH); NA (Not Available); A = blood pressure measurement; B = Head-Up Tilt Test (HUTT); C = The Orthostatic Hypotension Questionnaire (OHQ); D = Scales for Outcomes in Parkinson’s disease-Autonomic (SCOPA-AUT); E = PD Non-motor Symptom Questionnaire.

### 3.3 Risk of bias assessment of included studies

**Figure 2:**
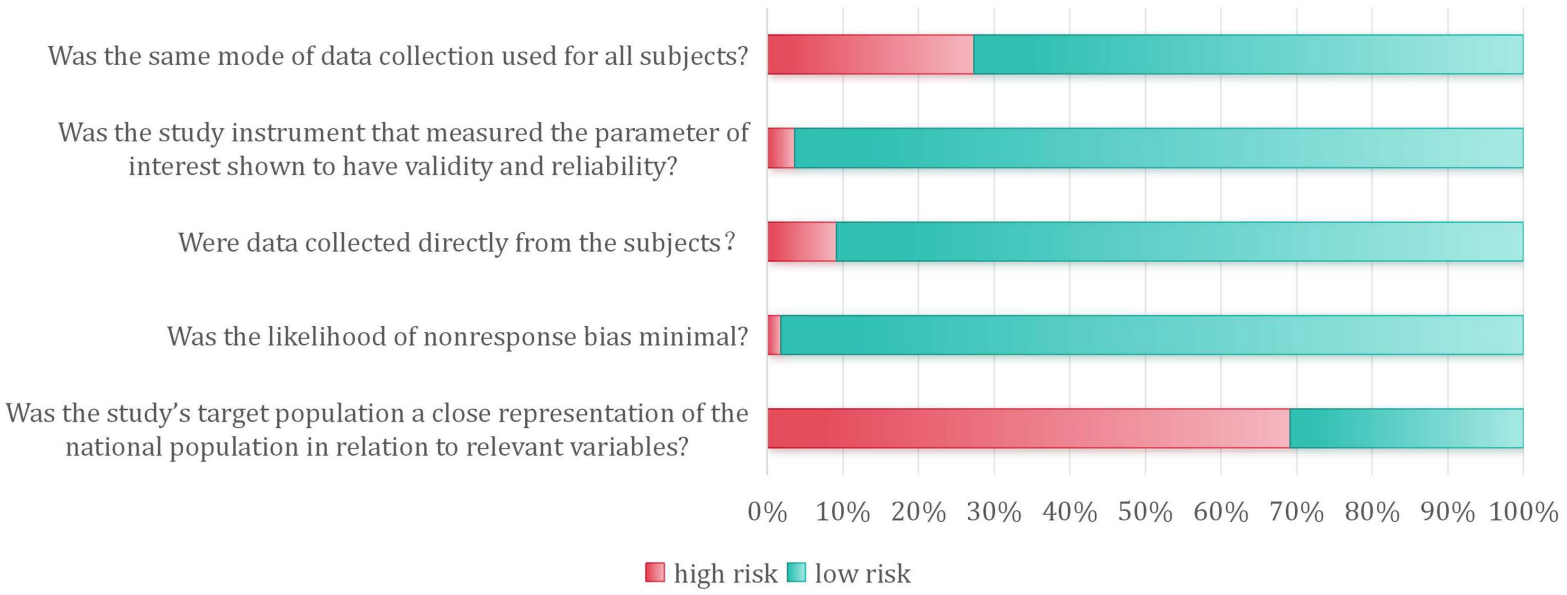
Partial bias risk assessment included in the study.

Risk of bias was assessed for all 55 included studies. In terms of external validity, key characteristics of the target population were explicitly described in 17 studies (31%). A sampling frame appropriate for representing the target population was employed in seven studies (13%); however, only one study was judged to have a truly representative sample. Participant recruitment achieved an ideal response rate (≥75%) in 54 studies (99%), while the response rate remained unclear for one study. Regarding internal validity, data were collected directly from participants in 50 studies (91%). All studies applied an acceptable definition of orthostatic hypotension, with 53 (96%) utilizing objective measurement methods. A standardized data collection protocol was employed across all participants in 40 studies (73%). All studies satisfied the criteria for adequate observation periods and correct numerator/denominator calculations. Ethical approval or informed consent was documented in all included studies. Detailed risk-of-bias assessments for each study are provided in Table 3, with a summary of the pooled results presented in Figure 2.

**Table 3:** Risk of bias assessment of the 55 full-text studies.

Author (year)	External validity				Internal validity						Total score
	1. Was the study’s target population a close representation of the national population in relation to relevant variables? (Yes/No)	2. Was the sampling frame a true or close representation of the target population? (Yes/No)	3. Was some form of random selection used to select the sample, OR, was a census undertaken? (Yes/No)	4. Was the likelihood of non-response bias minimal? (Yes/No)	5. Were data collected directly from the subjects (as opposed to a proxy)? (Yes/No)	6. Was an acceptable case definition used in the study? (Yes/No)	7. Was the study instrument that measured the parameter of interest shown to have reliability and validity (if necessary)? (Yes/No)	8. Was the same mode of data collection used for all subjects? (Yes/No)	9. Was the length of the shortest prevalence period for the parameter of interest appropriate? (Yes/No)	10. Were the numerator(s) and denominator(s) for the parameter of interest appropriate? (Yes/No)	
(34)	0	0	0	1	1	1	1	1	1	1	7
(35)	0	0	0	1	1	1	1	1	1	1	7
(36)	1	0	0	1	1	1	1	1	1	1	8
(37)	1	0	0	1	1	1	1	0	1	1	7
(38)	1	0	0	1	1	1	1	1	1	1	8
(39)	1	0	1	0	0	1	1	0	1	1	6
(40)	0	0	0	1	1	1	1	1	1	1	7
(41)	1	1	0	1	0	1	1	0	1	1	7
(42)	0	0	0	1	1	1	1	1	1	1	7
(43)	0	0	0	1	1	1	1	1	1	1	7
(44)	1	1	0	1	0	1	1	0	1	1	7
(45)	0	0	0	1	1	1	1	1	1	1	7
(46)	1	0	0	1	1	1	1	1	1	1	8
(47)	0	1	0	1	1	1	1	0	1	1	7
(48)	0	0	0	1	1	1	1	1	1	1	7
(20)	0	0	0	1	1	1	1	1	1	1	7
(49)	0	0	0	1	1	1	1	1	1	1	7
(50)	0	0	0	1	1	1	1	1	1	1	7
(51)	0	0	0	1	1	1	1	0	1	1	6
(52)	0	0	0	1	1	1	1	1	1	1	7
(53)	0	0	0	1	1	1	1	1	1	1	7
(54)	0	1	0	1	1	1	1	1	1	1	8
(55)	0	0	0	1	1	1	0	1	1	1	6
(56)	0	0	0	1	1	1	1	1	1	1	7
(57)	1	0	0	1	1	1	1	1	1	1	8
(58)	0	0	0	1	1	1	1	1	1	1	7
(59)	0	0	0	1	1	1	1	1	1	1	7
(60)	1	1	0	1	0	1	1	0	1	1	7
(61)	0	0	0	1	1	1	1	0	1	1	6
(62)	0	0	0	1	1	1	1	1	1	1	7
(63)	0	0	0	1	1	1	1	1	1	1	7
(64)	0	0	0	1	1	1	1	1	1	1	7
(65)	0	0	0	1	1	1	1	1	1	1	7
(66)	0	1	0	1	1	1	1	1	1	1	8
(67)	0	0	0	1	1	1	1	0	1	1	6
(68)	0	0	0	1	1	1	1	0	1	1	6
(69)	0	0	0	1	1	1	1	1	1	1	7
(70)	0	0	0	1	1	1	0	0	1	1	5
(71)	0	0	0	1	1	1	1	1	1	1	7
(72)	0	0	0	1	1	1	1	1	1	1	7
(73)	0	0	0	1	1	1	1	1	1	1	7
(74)	0	0	0	1	1	1	1	0	1	1	6
(75)	1	1	0	1	0	1	1	0	1	1	7
(76)	0	0	0	1	1	1	1	1	1	1	7
(77)	1	0	0	1	1	1	1	1	1	1	8
(78)	1	0	0	1	1	1	1	1	1	1	8
(79)	1	0	0	1	1	1	1	1	1	1	8
(80)	1	0	0	1	1	1	1	0	1	1	7
(81)	1	0	0	1	1	1	1	1	1	1	8
(82)	0	0	0	1	1	1	1	0	1	1	6
(18)	0	0	0	1	1	1	1	1	1	1	7
(83)	1	0	0	1	1	1	1	1	1	1	8
(84)	0	0	0	1	1	1	1	1	1	1	7
(85)	1	0	0	1	1	1	1	1	1	1	8
(86)	0	0	0	1	1	1	1	1	1	1	7

According to Hoy et al. (8), this risk of bias tool comprises 10 items plus a summary assessment. The item 1–4 assesses the external validity of the study (domains represent selection and nonresponse bias). The item 5–10 assesses the internal validity of the study (Item 5–9 assess domains of measurement bias, and item 10 assesses bias related to the analysis). Each item is answered as “Yes” (low risk, scored as 1 point), “No” (high risk, scored as 0 points), or “Uncertain” (reason must be explained, typically scored as 0 points).

### 3.4 Meta-analysis of the prevalence and incidence of orthostatic hypotension in patients with Parkinson’s disease

**Figure 3:**
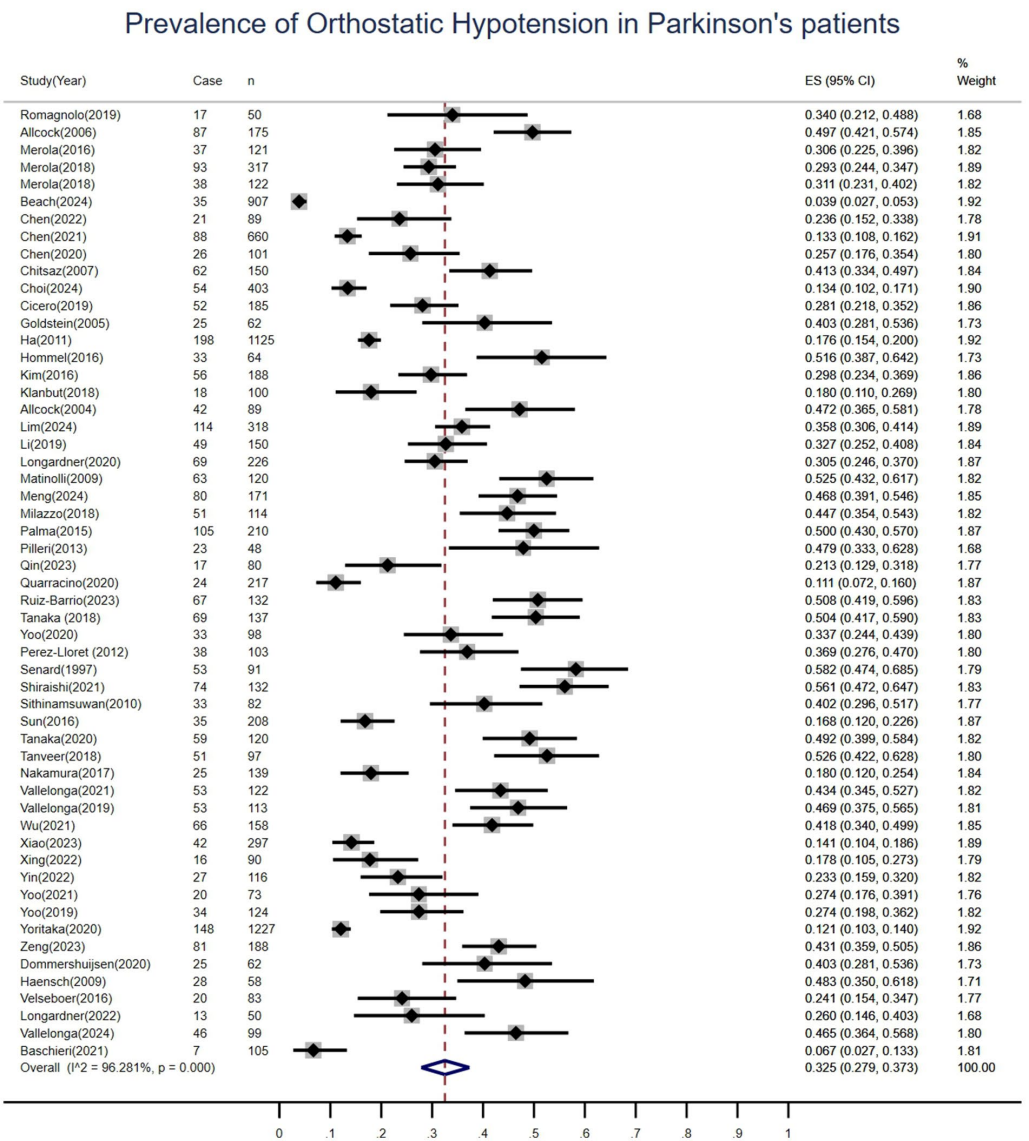
Forest plot for the pooled prevalence of orthostatic hypotension in patients with Parkinson’s disease.

This meta-analysis evaluated the prevalence of OH at multiple time points in patients with Parkinson’s disease, pooling data from 55 studies involving 10,463 patients. Among these, 2,793 cases of OH were identified, resulting in a pooled prevalence of 33% (95%*CI*: 28–37%), Figure 3. Significant heterogeneity was observed across studies (*I*^2^ = 96.3%, *p* < 0.01). Regarding OH incidence, only two prospective cohort studies provided extractable data, reporting incidence rates of 16 and 22%, respectively. In accordance with current meta-analysis guidelines requiring data from at least three studies for quantitative synthesis, pooling of incidence data was not performed.

### 3.5 Subgroup analyses

**Figure 4:**
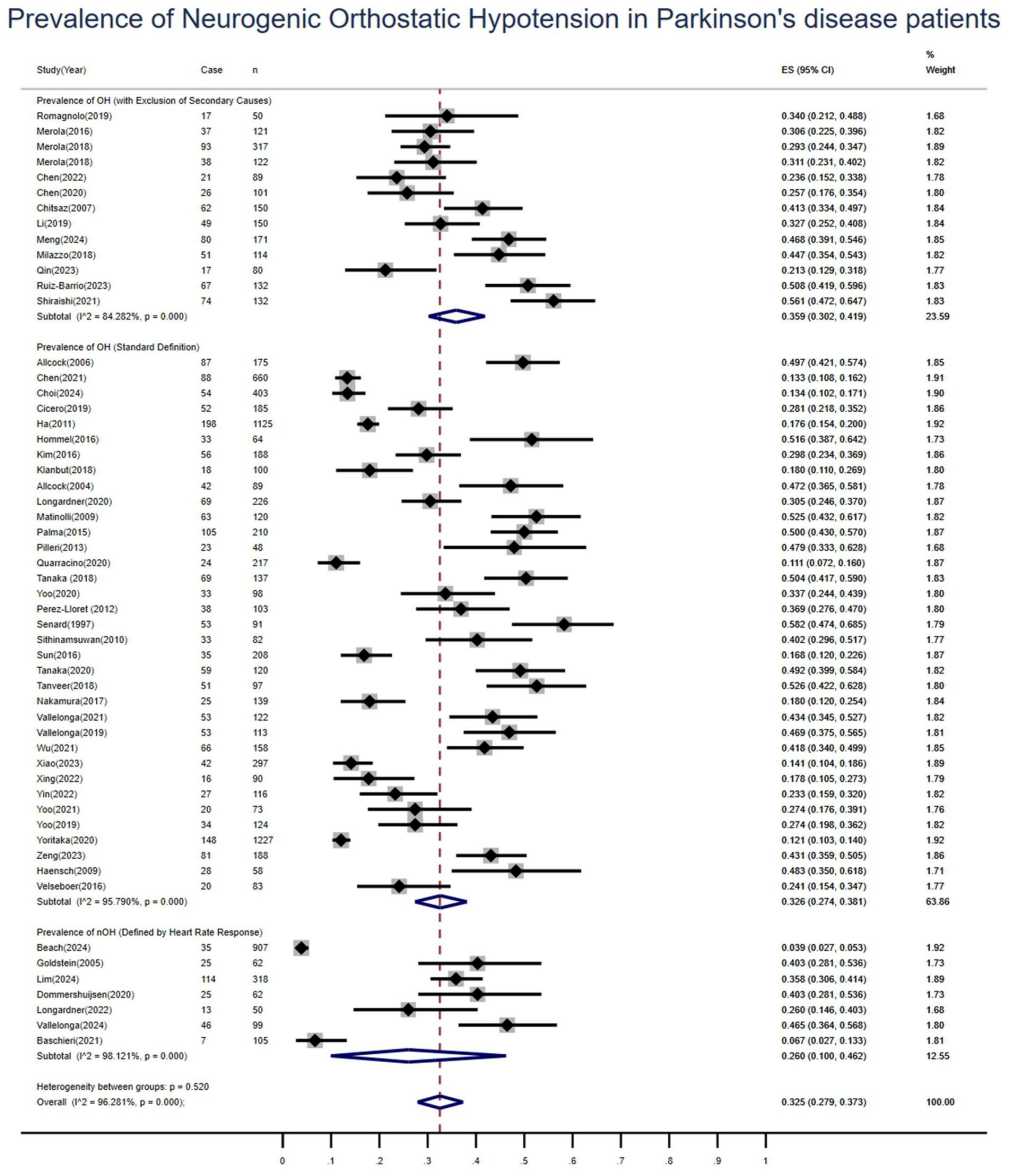
Forest plot of subgroup analyses for the pooled prevalence of neurogenic orthostatic hypotension in patients with Parkinson’s disease.

Among the 55 included studies, 35 adopted diagnostic criteria for OH based on consensus guidelines endorsed by international authorities: a sustained decrease of ≥20 mmHg in systolic blood pressure or ≥10 mmHg in diastolic blood pressure within 3 min of standing or tilting to at least 60°on a tilt table (13). Subgroup analyses were conducted based on these diagnostic definitions. The pooled prevalence of OH was 33% (95%*CI*: 27–38%; *I*^2^ = 96%). A sensitivity analysis, which excluded one study with unclear reporting and another that relied on subjective scales, was performed. The remaining 11 studies employing objective blood pressure measurements yielded a pooled OH prevalence of 33% (95%*CI*: 28–38%), with a substantial reduction in heterogeneity (*I*^2^ = 76%). A further subgroup analysis of 13 studies explicitly excluding patients with potential non-neurogenic causes of OH (e.g., diabetes, cancer, renal failure, stroke) showed a similar pooled prevalence of 36% (95% *CI*: 30–42%; *I*^2^ = 84.3%). Additionally, seven studies specifically reported on neurogenic orthostatic hypotension (nOH), defined by an impaired heart rate response to a drop in blood pressure, ΔHR/ΔSBP ratio <0.5 or a heart rate increase of <15 bpm (14). The pooled prevalence of nOH was 26% (95%*CI*: 10–46%; *I*^2^ = 98.1%), as detailed in Figure 4. To evaluate the robustness of this finding, a sensitivity analysis was conducted within the nOH subgroup. After excluding one study, the pooled prevalence based on blood pressure measurement criteria was 22% (95% *CI*: 10–38%; *I*^2^ = 96.6%).

Subgroup analyses were also performed according to symptomatic status. Based on 10 studies, the pooled prevalence of symptomatic orthostatic hypotension was 20% (95% *CI*: 13–29%; *I*^2^ = 90%). In contrast, based on six studies, the pooled prevalence of asymptomatic orthostatic hypotension was 22% (95% *CI*: 8–39%; *I*^2^ = 96%). Other results are presented in Table 4.

**Table 4:** Subgroup analysis results.

Subgroups	Studies	Prevalence	95% *CI*	*I*^2^ (%)	*p* Value
Gender_group					
Male	22	24	[19–28%]	90.9%	<0.001
Female	22	12	[10–14%]	65.6%	<0.001
Regions_group					
Europe	18	42%	[35–48%]	87.8%	<0.001
North America	4	28%	[19–38%]	92.8%	<0.001
Asian	24	32%	[26–37%]	94.8%	<0.001
mixed area	9	20%	[11–30%]	97.4%	<0.001
Published years (median)					
1997–2018	24	38%	[32–44%]	93.7%	<0.001
2019–2025	31	30%	[23–35%]	96.7%	<0.001
Sample size (median)					
*N* ≥ 121	28	30%	[24–36%]	97.5%	<0.001
*N* < 121	27	36%	[30–41%]	87.4%	<0.001
Diagnostic methods					
Head-up tilt test	10	28%	[19–37%]	91.8%	<0.001
Blood Pressure Measurement	43	33%	[28–38%]	96.6%	<0.001
Quality score					
High risk of bias	9	35%	[19–53%]	98.4%	<0.001
Moderate risk of bias	34	30%	[25–35%]	95.1%	<0.001
Low risk of bias	12	38%	[31–44%]	86.0%	<0.001

### 3.6 Regression analysis

To investigate the sources of the high heterogeneity observed across the 35 studies that employed the international diagnostic criteria for OH (13), we performed a meta-regression analysis. Although the association did not reach conventional statistical significance, the mean age of the patients emerged as the most influential covariate, accounting for a considerable portion of the between-study variance. For each one-year increase in mean age, the log-transformed OH prevalence increased by 0.056 units (95% *CI*: 0.013–0.125; *p* = 0.109). The between-study heterogeneity variance (*τ*^2^) decreased from 8.407 in the unadjusted model to 0.429 after adjusting for mean age, indicating that approximately 94.9% of the heterogeneity could be attributed to age (unadjusted *τ*^2^ = 8.407; adjusted *τ*^2^ = 0.429).

In contrast, meta-regression analyses based on the available data revealed no statistically significant associations between reported OH prevalence and disease duration (*β* = −0.029, *p* = 0.533; *R*^2^ = −3.74%), MDS-UPDRS- III score (*β* = 0.030, *p* = 0.251; *R*^2^ = 16.82%), or mean levodopa equivalent daily dose (*β* = 0.00043, *p* = 0.541; *R*^2^ = −4.39%). However, it must be emphasized that these findings are potentially limited by the considerable heterogeneity across studies, inconsistencies in variable definitions and measurement methods among the included studies, and the limited number of data points available for the regression analyses. Furthermore, a higher disease stage was observed to be positively associated with increased OH prevalence (*β* = 0.740, 95%*CI*: −0.085 to 1.566; *p* = 0.072). Although this association was not statistically significant, it explained approximately 36.7% of the heterogeneity. The statistical power of this analysis was limited due to the small number of studies available.

### 3.7 Publication bias analysis

**Figure 5:**
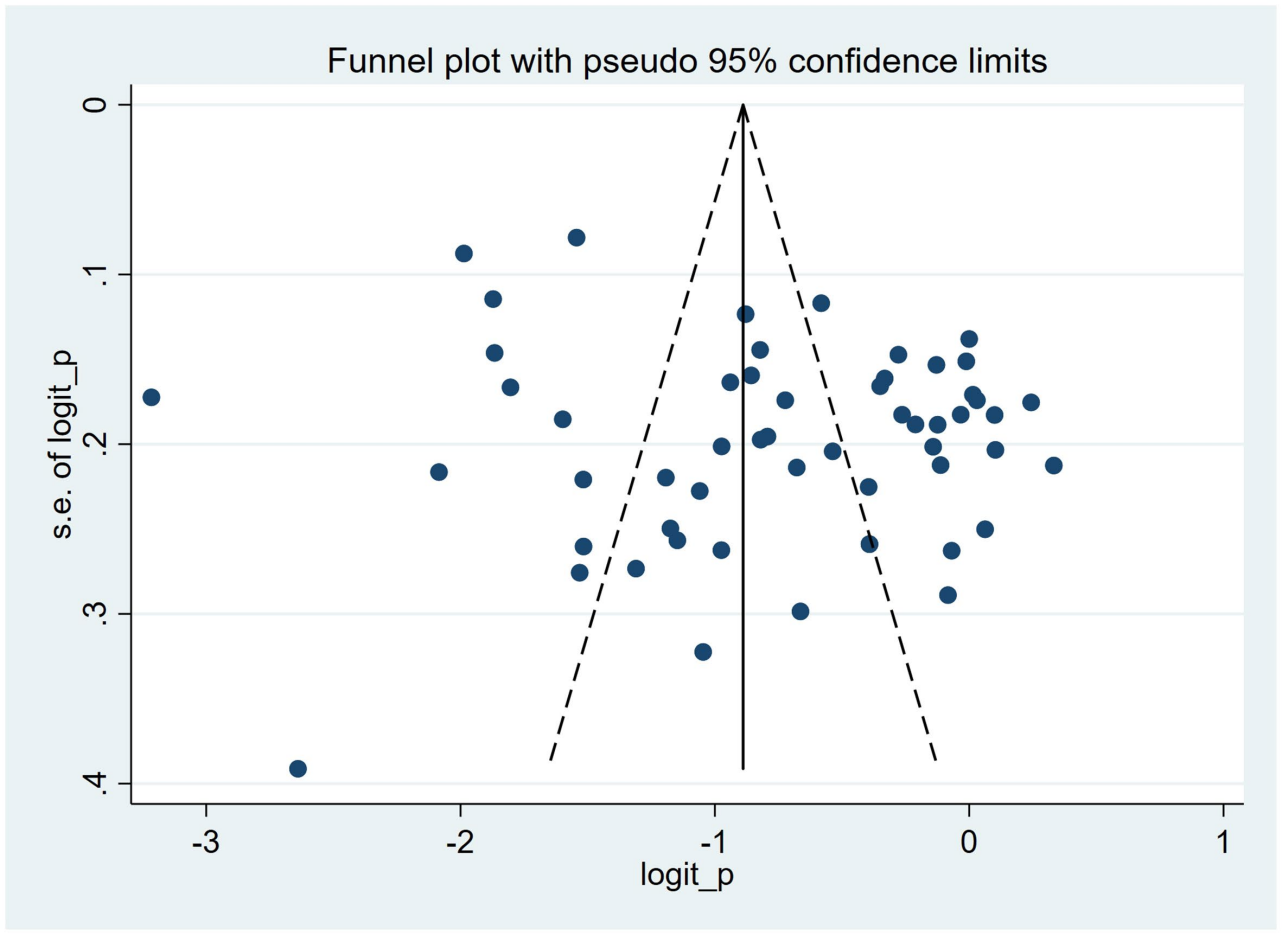
Funnel plot evaluating publication bias across included studies (*n*=55).

The funnel plot exhibited asymmetry, which may reflect the presence of publication bias across the 55 studies (Figure 5). Quantitative evidence from Egger’s test showed a statistically significant small-study effect (intercept = 4.38, 95% CI 0.69–8.06, *p* = 0.021). Applying the trim-and-fill method to address this asymmetry involved imputing 22 studies. Consequently, the pooled prevalence estimate adjusted from 33.0% (95% CI 29.0–37.0%) to 24.9% (95% CI 20.0–31.1%), indicating that the initial finding might have been overestimated due to the potential exclusion of smaller or non-significant studies.

## 4 Discussion

### 4.1 Principal findings

This meta-analysis, encompassing 55 studies and 10,463 patients with PD, yielded a pooled prevalence of OH of 33%. This finding underscores the high frequency of OH in PD and confirms its role as a clinically significant comorbidity in disease management. OH is a core manifestation of autonomic dysfunction and has been consistently linked to an elevated risk of falls, cognitive decline, diminished quality of life, and increased cardiovascular morbidity (15). Consequently, our results strongly advocate for the integration of routine OH screening into standard follow-up assessments for PD patients to facilitate early detection and intervention. Compared with earlier meta-analytic estimates (5), the present study provides a more precise prevalence estimate based on a substantially larger sample size, thereby strengthening the evidence on the disease burden attributable to OH in PD.

Significant heterogeneity existed among studies (*I*^2^ = 96.3%, *p* < 0.01), indicating that pooled estimates were influenced by a combination of clinical and methodological factors. Potential sources of clinical heterogeneity included patient age and Horn-Yar staging. Furthermore, differences in the proportion of neurogenic versus non-neurogenic OH across study populations may contribute to heterogeneity in prevalence estimates. Methodological heterogeneity primarily stems from inconsistencies in diagnostic criteria and assessment techniques. Current international consensus guidelines recommend diagnosing nOH based on direct or indirect assessment of sympathetic vasomotor function, rather than relying solely on ratios such as ΔHR/ΔSBP (16). However, most studies included in this meta-analysis employed only hemodynamically defined nOH criteria and did not systematically apply or report comprehensive autonomic function assessments to distinguish nOH. This widespread lack of systematic assessment introduces etiological confounding into prevalence estimates of OH and may limit in-depth analysis of OH associations with specific Parkinson’s disease factors (e.g., levodopa equivalent dose or MDS-UPDRS-III score). Furthermore, measurement bias may arise as some studies employed active supine-to-standing blood pressure measurement, while others relied on self-reported symptoms or retrospective data. Future prospective studies should employ standardized autonomic testing protocols to more clearly define neurogenic orthostatic hypotension in Parkinson’s disease.

Notably, only two prospective cohort studies provided extractable incidence data, reporting rates of 16 and 22%, respectively. In accordance with current meta-analysis guidelines, which typically require data from at least three studies for quantitative synthesis, a pooled analysis of incidence rates was not feasible. This limitation highlights a significant lack of high-quality prospective evidence in this area. Future research should prioritize well-designed, longitudinal cohort studies that employ standardized protocols for assessing OH, in order to accurately determine its annual incidence and cumulative risk in patients with Parkinson’s disease.

### 4.2 Subgroup analysis and meta-regression findings

This meta-analysis systematically evaluated the prevalence of OH in patients with PD. The pooled results revealed a combined prevalence of 33% according to international consensus criteria, confirming that OH is a common and significant non-motor symptom in the PD population (17). More importantly, even after excluding studies with apparent non-neurogenic confounding factors, the prevalence remained as high as 36%, providing robust evidence that inherent autonomic dysfunction in PD is a key pathophysiological basis for OH development. OH in PD exhibits significant heterogeneity, manifesting in both pathophysiological and clinical dimensions. Pathophysiologically, the prevalence of strictly defined nOH, characterized by an impaired heart rate response, was 26%. This indicates that a considerable proportion of patients meeting the traditional blood pressure criteria for OH may not have complete failure of cardiovascular autonomic reflexes, possibly retaining partial compensatory mechanisms or being confounded by reversible factors such as medications or hypovolemia (18). The extremely high heterogeneity observed in this subgroup (*I*^2^ = 98%) indicates that current assessments of nOH lack standardization in measurement timing (e.g., medication-related), diagnostic criteria, and environmental controls. This constitutes a key methodological issue requiring resolution in future research. Clinically, the prevalence of symptomatic OH (20%) is comparable to that of asymptomatic OH (22%). However, the presence of asymptomatic OH indicates that reliance on clinical symptoms alone leads to underdiagnosis. Studies have confirmed that asymptomatic OH is also associated with an increased risk of falls, cognitive decline, and cardiovascular events (19). Therefore, implementing standardized supine-to-standing blood pressure screening for all PD patients, rather than relying solely on symptom reporting, is a necessary clinical practice supported by clear evidence.

The pooled prevalence estimates in this study were significantly influenced by various methodological and population factors. First, the OH prevalence measured using conventional sphygmomanometers (33%) was higher than that obtained via head-up tilt testing (28%), suggesting that measurement tools must be considered when comparing or integrating data from different studies (20). Second, sample size may introduce bias, with smaller studies (*N* < 121) showing a slightly higher prevalence, indicating potential small-study effects or publication bias. Regarding demographic characteristics, the male prevalence rate (24%) was significantly higher than that of females (12%). This finding aligns with the conclusion of a study (21), which reported no statistically significant difference. This disparity may be attributed to the overall gender composition of the included populations, fluctuations in sex hormone levels, and gender-specific developmental differences in central dopaminergic circuits. Future well-designed prospective studies with subgroup analyses are needed to validate whether gender constitutes an independent risk factor. In terms of regional differences, studies from Europe reported the highest prevalence (42%), followed by Asia (32%), with mixed regions showing a relatively lower prevalence. These disparities more likely reflect imbalances in the emphasis on autonomic function assessment, diagnostic awareness, and accessibility of healthcare resources in neurological clinical practices across different global regions. Furthermore, the lack of data from Oceania and Africa in this analysis limits the global representativeness of the conclusions and underscores the urgency of conducting epidemiological surveys in these regions, where healthcare and research resources may be relatively scarce, to clarify the disease burden (22, 23). Additionally, temporal trend analysis showed that the pooled OH prevalence in more recent studies (2019–2025) was lower (30%) than in earlier studies (1997–2018, 38%). This decline is likely attributable to the continuous updates and clinical dissemination of numerous guidelines and consensuses on OH management in PD patients over the past decade (24–26), which have enhanced healthcare professionals’ awareness and capability for early identification, prevention, and management. Concurrently, the promotion of patient education and the of non-pharmacological interventions may have played a crucial role (27, 28).

In summary, this study not only quantified the high prevalence of OH in PD patients but also provided an in-depth exploration of its inherent heterogeneity and the external factors influencing prevalence estimates. It is strongly recommended to integrate standardized orthostatic blood pressure assessment into the routine management of PD patients and to establish a standardized diagnostic for nOH, including measurement conditions. Future research should aim to clarify risk factors such as gender and region through prospective designs and continuously evaluate the long-term impact of clinical guideline implementation and practice improvements on OH prevalence.

### 4.3 Comparison with other studies

This study builds upon and extends the findings of a prior systematic review by Velseboer et al. (5), which reported a pooled OH prevalence of 30.4% across 25 studies up to 2009. Our updated meta-analysis, incorporating more recent literature, refines the global epidemiological profile of OH in PD. Notably, we performed novel subgroup and meta-regression analyses to investigate potential sources of heterogeneity related to etiology, measurement methodology, and key clinical variables, including age, disease duration, and stage.

Meta-regression analysis revealed that the mean age of patients played a pivotal role in explaining the substantial heterogeneity observed across studies. Although the association between mean age and the log-transformed prevalence did not reach conventional statistical significance (*p* = 0.109), model fit provided compelling evidence: adjusting for age alone reduced the between-study heterogeneity variance (*τ*^2^) by approximately 94.9% (from 8.407 to 0.429). This indicates that differences in the mean age of study cohorts are the primary driver of the wide variability in reported orthostatic hypotension prevalence. This finding aligns with the understanding that orthostatic hypotension in Parkinson’s disease results from the confluence of disease-specific autonomic dysfunction and age-related physiological decline in systemic autonomic function and vascular compliance (29). Consequently, advanced age should be considered a paramount risk indicator for orthostatic hypotension in Parkinson’s disease, informing future primary prevention strategies and clinical risk stratification.

In contrast, other clinical variables demonstrated limited explanatory power. Neither disease duration, motor symptom severity (MDS-UPDRS-III score), nor average daily levodopa-equivalent dose showed significant association with OH prevalence, and incorporating these variables contributed minimally to reducing heterogeneity. Although dopaminergic medications are widely recognized as potential triggers or exacerbators of OH (30), their effect size was not a primary source of inter-study variation in this meta-analysis. This may be attributed to common clinical practices, such as proactive dose adjustments or non-pharmacological interventions in high-risk patients, which likely attenuated observable population-level effects. Furthermore, critical methodological flaws obscure potential true associations: most included studies failed to standardize or report the timing relationship between blood pressure measurements and medication administration. Research confirms levodopa as an acute hypotensive agent, with the highest risk observed in patients with pre-existing autonomic dysfunction (31). Our meta-analysis, however, reveals that in long-term population-level observations, the complex interplay of clinical adaptations, prescribing strategies, and disease progression confounds the simple linear relationship between LEDD and OH prevalence, rendering it non-significant. This underscores the need for future studies to meticulously document medication timing, OH measurement timing, and differentiate nOH to more accurately elucidate the relationship between medication and autonomic complications.

The meta-regression analysis in this study revealed a strong positive correlation trend between Hoehn & Yahr disease staging and the prevalence of OH, explaining approximately 36.7% of the heterogeneity among studies, although this association did not reach conventional statistical significance (*p* = 0.072). Recent studies confirm that sympathetic dysfunction can be detected through sophisticated autonomic testing in early-stage PD patients before clinical OH manifests (32). This suggests that OH, as a clinical diagnosis, may lag behind initial autonomic pathological damage and represents a state of partial decompensation. Therefore, the timing of OH screening in PD patients will become a critical phase for precision management.

The risk of bias assessment conducted in this review highlights critical methodological limitations that should be addressed in future research. First, the prevalence of orthostatic hypotension must be assessed using an internationally recognized operational definition specific to Parkinson’s disease (33). Most of the included studies did not specify key methodological details, such as the timing of assessments in relation to medication intake or patients’ medication status, complicating cross-study comparisons and pooled analyses. Second, while this review intended to explore potential differences in OH incidence and prevalence between urban and rural populations, the available literature did not stratify or explicitly report the geographic setting of study populations. Future studies with expanded sample sizes are recommended to collect and report such data. This would greatly assist clinicians in understanding the potential socio-environmental influences on OH epidemiology and inform tailored treatment and intervention strategies.

### 4.4 Research limitations and implications

The strengths of this study lie in its rigorous systematic review methodology and comprehensive reporting. We also deduplicated data reported repeatedly across multiple studies by the same authors, integrated the most comprehensive dataset, and updated previously published research. However, we acknowledge several limitations. Due to resource constraints, we included only studies published in English, potentially introducing publication bias. Secondly, the pooled data exhibited high heterogeneity. Nevertheless, we attempted to explain clinical and methodological differences through meta-regression analysis and subgroup analyses. Pooled results report confidence intervals (CI). Furthermore, due to unclear reporting in original studies or incompatible data types, the number of subgroup analyses for disease duration, disease staging, and other factors was limited, potentially introducing bias in their results. This underscores the need for future studies to comprehensively collect patient disease characteristics and thoroughly investigate the prevalence and incidence of orthostatic hypotension caused by different etiologies. Such research will provide a reference basis for clinical practitioners in preventing and managing orthostatic hypotension.

## 5 Conclusion

Orthostatic hypotension (OH) exhibits a high prevalence in patients with Parkinson’s disease, although reported estimates demonstrate substantial variability across studies. This heterogeneity largely arises from considerable differences in clinical characteristics and methodological approaches. While our analysis did not establish statistically significant associations between OH prevalence and disease duration, MDS-UPDRS-III score, or levodopa dose at the meta-analytic level, these findings likely reflect inconsistencies in assessment methods and case definitions across existing observational studies rather than negating a pathophysiological role for these factors at the individual patient level. Future research must prioritize the adoption of standardized autonomic function assessment protocols, as recommended by international consensus, to more precisely define neurogenic OH. Employing such standardized criteria will be crucial for delineating clear risk factors, elucidating the natural history, and determining associations with antiparkinsonian therapies within well-characterized, homogeneous cohorts, thereby generating more robust evidence to inform clinical management.

## Data Availability

The original contributions presented in the study are included in the article/Supplementary material, further inquiries can be directed to the corresponding author.
