# Supplementary material for: Prevalence and incidence of orthostatic hypotension in patients with Parkinson’s disease: an updated systematic review and meta-analysis
Source: Front Neurol. 2026 Feb 12;17:1751756. doi: 10.3389/fneur.2026.1751756 (PMC12935681; doi:10.3389/fneur.2026.1751756)
Supplement: Supplementary file 1 [file Supplementary_file_1.docx]

**Appendix 1: Excluded studies and reasons (N = 188)**

Through systematic database searches, we identified 8,106 records. Manual searches of reference lists identified an additional 3 records. After removing duplicates, 3,714documents remained. After preliminary review of titles and abstracts, 3,471 documents were excluded, leaving 243documents.

| No. | author | year | title | Reasons for exclusion |
| --- | --- | --- | --- | --- |
|  | Li, M  Zhou, L  Ma, J | 2024 | [Acupuncture at stellate ganglion combined with western medication for orthostatic hypotension in Parkinson's disease: a randomized controlled trial] | Study type mismatch: Randomized controlled trial |
|  | Wöber-Bingöl, CWessely, P  Gradner, W  Deecke, L | 1990 | [24-hour blood pressure determination in Shy-Drager syndrome. A case report] | Study population mismatch：Shy-Drager syndrome |
|  | Saito, F  Tsuchiya, K  Kotera, M | 1992 | [An autopsied case of Parkinson's disease manifesting Shy-Drager syndrome] | Study population mismatch：Shy-Drager syndrome |
|  | Furgała, A  Górecka-Mazur, A  Fiszer, U  Pietraszko, | 2015 | [Evaluation of heart rate and blood pressure variability in Parkinson's disease patients after bilateral subthalamic deep brain stimulation] | Study type mismatch: Randomized controlled trial |
|  | Shadmand, M  Elliott, B  Lautze, J | 2024 | A retrospective analysis of neurogenic orthostatic hypotension in long-term care facility residents with recurrent falls | Study population mismatch：Residents who fall repeatedly |
|  | Liu, Z  Su, D | 2023 | Acute effect of levodopa on orthostatic hypotension and its association with motor responsiveness in Parkinson's disease: Results of acute levodopa challenge test | Study type mismatch: Randomized controlled trial |
|  | Liu, Z  Su, D  Zhou, J  Wang, X | 2023 | Acute effect of levodopa on orthostatic hypotension and its association with motor responsiveness in Parkinson's disease: Results of acute levodopa challenge test | Study type mismatch: Randomized controlled trial |
|  | Hoxhaj, P  Shah, S  Muyolema, Arce VE  Gupta, I | 2023 | Ampreloxetine Versus Droxidopa in Neurogenic Orthostatic Hypotension: A Comparative Review | Study type mismatch: Review |
|  | Li, Z  Jiang, X  Yang, M | 2023 | Association between falls and nonmotor symptoms in patients with Parkinson's disease | Outcome measure discrepancy：fall |
|  | Kim, J S  Oh, Y S  Lee, K S | 2012 | Association of cognitive dysfunction with neurocirculatory abnormalities in early Parkinson disease | Outcome measure discrepancy：cognitive impairment |
|  | Mu, F  Jiao, Q  "Du X" | 2020 | Association of orthostatic hypotension with Parkinson's disease: a meta-analysis | Study type mismatch: Meta-analysis |
|  | Mathias, C J | 1998 | Cardiovascular autonomic dysfunction in parkinsonian patients | Study type mismatch: Review |
|  | Khalil, I  Sayad, R | 2024 | Cardiovascular dysautonomia and cognitive impairment in Parkinson's disease (Review) | Study type mismatch: Review |
|  | Nascimento, D | 2021 | Clinical features associated with drooling in Parkinson's disease | Outcome measure discrepancy：Saliva characteristics |
|  | Riley, D E  Espay, A J | 2018 | Cognitive fluctuations in Parkinson's disease dementia: blood pressure lability as an underlying mechanism | Study population mismatch：Parkinson's dementia patients |
|  | Smith, G D  Mathias, C J | 1996 | Differences in cardiovascular responses to supine exercise and to standing after exercise in two clinical subgroups of Shy-Drager syndrome (multiple system atrophy) | Study population mismatch：Shy-Drager syndrome |
|  | He, X  Mo, C  Zhang, Y | 2021 | Effect of Acute Levodopa Up-Titration on Blood Pressure in Patients With Early Stage Parkinson's Disease: Results of a Levodopa Challenge Test | Study type mismatch: Randomized controlled trial |
|  | Earl, T  Jridi, A  Thulin, P C | 2024 | Effect of levodopa on postural blood pressure changes in Parkinson disease: a randomized crossover study | Study type mismatch: Randomized controlled trial |
|  | Su, D  Zhang, X  Su, Y | 2023 | Effects of different levodopa doses on blood pressure in older patients with early and middle stages of Parkinson's disease | Study type mismatch: experimental research |
|  | Rascol, O  Perez-Lloret, S  Damier, P | 2015 | Falls in ambulatory non-demented patients with Parkinson's disease | Outcome measure discrepancy：fall |
|  | Michałowska, M  Fiszer, U  Krygowska-Wajs, A  Owczarek, K | 2005 | Falls in Parkinson's disease. Causes and impact on patients' quality of life | Outcome measure discrepancy：fall |
|  | Friedman, J H  Abrantes, A | 2011 | Fatigue in Parkinson's disease | Outcome measure discrepancy：fatigue |
|  | Montastruc, J L  Pelat, M | 1998 | Fluoxetine in orthostatic hypotension of Parkinson's disease: a clinical and experimental pilot study | Study type mismatch: experimental research |
|  | Elliott, J E  Bryant-Ekstrand, M | 2023 | Frequency of Orthostatic Hypotension in Isolated REM Sleep Behavior Disorder | Study population mismatch：Patients with sleep behavior disorders |
|  | Pathak, A  Lapeyre-Mestre, M  Montastruc, J L  Senard, J M | 2005 | Heat-related morbidity in patients with orthostatic hypotension and primary autonomic failure | Study population mismatch：Patients with autonomic dysfunction receiving drug treatment |
|  | Roberson, K B  Jacobs, K A  Eltoukhy, M | 2019 | Hemodynamic responses to an exercise stress test in Parkinson's disease patients without orthostatic hypotension | Study type mismatch: experimental research |
|  | Poon, I O  Braun, U | 2005 | High prevalence of orthostatic hypotension and its correlation with potentially causative medications among elderly veterans | Study population mismatch：Military personnel aged 75 and above |
|  | Jones, J D  Jacobson, C  Murphy, M | 2014 | Influence of hypertension on neurocognitive domains in nondemented Parkinson's disease patients | Outcome measure discrepancy：Hypotension/Hypertension |
|  | Sturchio, A  Dwivedi, A K  Marsili, L  Hadley, A | 2021 | Kinematic but not clinical measures predict falls in Parkinson-related orthostatic hypotension | Outcome measure discrepancy：fall |
|  | Senard, J M  Rascol, O  Rascol, A  Montastruc, J L | 1993 | Lack of yohimbine effect on ambulatory blood pressure recording: a double-blind cross-over trial in parkinsonians with orthostatic hypotension | Study type mismatch: experimental research |
|  | Mathias, C J | 2008 | L-dihydroxyphenylserine (Droxidopa) in the treatment of orthostatic hypotension: the European experience | Study type mismatch: experimental research |
|  | Stanková, S  Straka, I  Košutzká, Z | 2022 | Levodopa-Carbidopa Intestinal Gel Improves Symptoms of Orthostatic Hypotension in Patients with Parkinson's Disease-Prospective Pilot Interventional Study | Study type mismatch: experimental research |
|  | Castro, R A  Vernetti, P M | 2023 | Long-Term Outcomes of Hyperadrenergic Orthostatic Hypotension | Study type mismatch: experimental research |
|  | Miller, RJH  Chew, D S | 2019 | Neglected cause of recurrent syncope: a case report of neurogenic orthostatic hypotension | Study type mismatch: Case report |
|  | Espay, A J  LeWitt, P A  Hauser, R A | 2016 | Neurogenic orthostatic hypotension and supine hypertension in Parkinson's disease and related synucleinopathies: prioritisation of treatment targets | Study type mismatch: Review |
|  | DiFrancisco-Donoghue, J | 2019 | Nicotine Gum as a Therapeutic Approach for Low Blood Pressure in Parkinson's Disease: A Randomized Pilot Study | Outcome measure discrepancy：experimental research |
|  | Mussi, C  Ungar, A  Salvioli, G | 2009 | Orthostatic hypotension as cause of syncope in patients older than 65 years admitted to emergency departments for transient loss of consciousness | Study population mismatch：Syncope patient |
|  | Ahn, J H  Cho, J W  Youn, J | 2023 | Orthostatic Hypotension Is a Predictor of Fatigue in Drug-Naïve Parkinson's Disease | Outcome measure discrepancy：fatigue |
|  | Freeman, R  Abuzinadah, A R  Gibbons, C | 2018 | Orthostatic Hypotension: JACC State-of-the-Art Review | Study type mismatch: Review |
|  | Kincl, V  Panovský, R  Bočková, M | 2024 | Parkinson´s disease cardiovascular symptoms: A new complex functional and structural insight | Outcome measure discrepancy:Heart rate and blood pressure |
|  | Montastruc, J L  Chamontin, B  Rascol, A | 1985 | Parkinson's disease and hypertension: chronic bromocriptine treatment | Study type mismatch: experimental research |
|  | Pathak, A  Senard, J M | 2004 | Pharmacology of orthostatic hypotension in Parkinson's disease: from pathophysiology to management | Study type mismatch: Review |
|  | Pavelić, A  Krbot, Skorić M | 2017 | Postprandial hypotension in neurological disorders: systematic review and meta-analysis | Outcome measure discrepancy:postprandial hypotension |
|  | Hamad, A  Hussain, M E | 2019 | Prevalence and Management of Hypokalemia in Peritoneal Dialysis Patients in Qatar | Outcome measure discrepancy:hypokalemia |
|  | L, E A  C, A A | 2011 | Prevalence of orthostatic hypotension in a series of elderly Mexican institutionalized patients | Study population mismatch：墨Elderly people in Xigo |
|  | Velseboer, D C  de Haan, R J  Wieling, W | 2011 | Prevalence of orthostatic hypotension in Parkinson's disease: a systematic review and meta-analysis | Study type mismatch: Systematic review |
|  | McDonagh, STJ  Mejzner, N  Clark, C E | 2021 | Prevalence of orthostatic hypotension in primary, community and institutional care: a systematic review and meta-analysis | Study population mismatch：Community institutions and groups |
|  | Biswas, D  Karabin, B  Turner, D | 2019 | Role of nurses and nurse practitioners in the recognition, diagnosis, and management of neurogenic orthostatic hypotension: a narrative review | Study type mismatch: review |
|  | Becerra, A F  Boch, M | 2021 | Ropinirole-Associated Orthostatic Hypotension as Cause of a Prescribing Cascade in an Elderly Man | Study population mismatch：elderly people |
|  | Isaacson, S  Shill, H A  Vernino, S | 2016 | Safety and Durability of Effect with Long-Term, Open-Label Droxidopa Treatment in Patients with Symptomatic Neurogenic Orthostatic Hypotension (NOH303) | Study type mismatch: experimental research |
|  | Hattori, N  Kajita, M  Fujimoto, S | 2024 | Safety and effectiveness of rasagiline in patients with Parkinson's disease in Japan: a post-marketing surveillance study | Study type mismatch: experimental research |
|  | Bhattacharya, K F  Nouri, S | 2003 | Selegiline in the treatment of Parkinson's disease: its impact on orthostatic hypotension | Study type mismatch: experimental research |
|  | Fanciulli, A  Göbel, G | 2016 | Supine hypertension in Parkinson's disease and multiple system atrophy | Outcome measure discrepancy:supine hypertension |
|  | Isik, Ahmet Turan  Kocyigit, Suleyman Emre | 2019 | A comparison of the prevalence of orthostatic hypotension between older patients with Alzheimer's Disease, Lewy body dementia, and without dementia | Study population mismatch：Alzheimer's disease |
|  | Loureiro, Debora  Bilbao, Rodrigo  Bordet, Sofia  Grasso, Lina | 2023 | A systematic review and meta-analysis on the association between orthostatic hypotension and mild cognitive impairment and dementia in Parkinson's disease | Study type mismatch: systematic review |
|  | Liu, Zhu  Su, Dongning  Zhou, Junhong  Wang, Xuemei | 2023 | Acute effect of levodopa on orthostatic hypotension and its association with motor responsiveness in Parkinson's disease: Results of acute levodopa challenge test | Study type mismatch: experimental research |
|  | Kwon, Kyum-Yil  Park, Suyeon  Lee, Mina  Ju, Hyunjin | 2020 | Dizziness in patients with early stages of Parkinson's disease: Prevalence, clinical characteristics and implications | Outcome measure discrepancy:Syncope patient |
|  | Bacchi, Stephen  Chim, Ivana  Kramer, Philippe | 2017 | Domperidone for Hypotension in Parkinson's Disease: A Systematic Review | Study type mismatch: Systematic review |
|  | Earl, Timi  Jridi, Amani | 2024 | Effect of levodopa on postural blood pressure changes in Parkinson disease: a randomized crossover study | Study type mismatch: randomized controlled trial |
|  | Paschen, S  Hansen, C  Welzel, J | 2022 | Effect of lower limb vs. abdominal compression on mobility in orthostatic hypotension - a single-blinded, randomized, controlled, cross-over study in Parkinson's disease | Study type mismatch: Randomized controlled trial |
|  | Rascol, Olivier  Perez-Lloret, | 2015 | Falls in ambulatory non-demented patients with Parkinson's disease | Outcome measure discrepancy:fall |
|  | Pelicioni, Paulo H S  Menant, Jasmine C | 2019 | Falls in Parkinson's Disease Subtypes: Risk Factors, Locations and Circumstances | Outcome measure discrepancy:fall |
|  | Michatowska, M  Fiszer, U | 2005 | Falls in Parkinson's disease. Causes and impact on patients' quality of life | Outcome measure discrepancy:fall |
|  | Friedman, Joseph H  Abrantes, Ana | 2011 | Fatigue in Parkinson's disease | Outcome measure discrepancy:fatigue |
|  | Kotagal, Vikas  Szpara, Ashley | 2019 | Fatigue in Parkinson's Disease Associates with Lower Ambulatory Diastolic Blood Pressure | Outcome measure discrepancy:fatigue |
|  | Veazie, Stephanie  Peterson, Kim | 2021 | Fludrocortisone for orthostatic hypotension | Study type mismatch: Randomized controlled trial |
|  | Wojszel, Z B  Kasiukiewicz, A  Magnuszewski, L | 2019 | Health and Functional Determinants of Orthostatic Hypotension in Geriatric Ward Patients: A Retrospective Cross Sectional Cohort Study | Study population mismatch：elderly people |
|  | Gallop, Katy  Pham, Ngan  Maclaine, Grant | 2023 | Health related quality-of-life and burden for caregivers of individuals with neurogenic orthostatic hypotension | Study population mismatch：nurses |
|  | Roberson, Kirk B  Signorile, Joseph F  Singer, Carlos | 2019 | Hemodynamic responses to an exercise stress test in Parkinson's disease patients without orthostatic hypotension | Outcome measure discrepancy:Pulse rate |
|  | Poon, I O  Braun, U | 2005 | High prevalence of orthostatic hypotension and its correlation with potentially causative medications among elderly veterans | Study population mismatch：elderly veterans |
|  | Cronin, Padraig  Collins, Lucy M | 2024 | Impacts of gait freeze on quality of life in Parkinson's disease, from the perspectives of patients and their carers | Outcome measure discrepancy:quality of life |
|  | Winkler, A S | 2001 | Investigation of the pathogenesis of anaemia and symptomatic orthostatic hypotension in diabetic autonomic neuropathy and multiple system atrophy: use of a novel treatment. (BL: DXN054257) | Study population mismatch：diabetic patient |
|  | Sturchio, Andrea  Dwivedi, Alok K  Marsili, Luca  Hadley, Aaron | 2021 | Kinematic but not clinical measures predict falls in Parkinson-related orthostatic hypotension | Outcome measure discrepancy:fall |
|  | Senard, J M  Rascol, O  Rascol, A | 1993 | Lack of yohimbine effect on ambulatory blood pressure recording: a double-blind cross-over trial in parkinsonians with orthostatic hypotension. | Study type mismatch: randomized controlled trial |
|  | Maule, Simona  Milazzo, Valeria  Maule, Milena Maria | 2012 | Mortality and prognosis in patients with neurogenic orthostatic hypotension | Study population mismatch：Patients with neurogenic orthostatic hypotension |
|  | JAIN, SAMAY | 2011 | Multi-Organ Autonomic Electrophysiology in Parkinson Disease | Outcome measure discrepancy:motor symptoms |
|  | Miller, Robert J H  Chew, Derek S  Raj, Satish R | 2019 | Neglected cause of recurrent syncope: a case report of neurogenic orthostatic hypotension | Study type mismatch: case report |
|  | Turana, Yuda  Shen, Robert | 2022 | Neurodegenerative diseases and blood pressure variability: A comprehensive review from HOPE Asia | Study type mismatch: review |
|  | Rass, Verena  Beer, Ronny  Schiefecker, Alois J | 2021 | Neurological outcome and quality of life 3 months after COVID-19: A prospective observational cohort study | Study population mismatch：COVID-19 patients |
|  | Biaggioni, Italo | 2014 | New Developments in the Management of Neurogenic Orthostatic Hypotension | Study type mismatch: review |
|  | Traon, A Pavy-Le  Piedvache, A | 2016 | New insights into orthostatic hypotension in multiple system atrophy: a European multicentre cohort study | Study population mismatch：multiple system atrophy patient |
|  | Ling, Yu-Ting  Guo, Qian-Qian  Wang, Si-Min | 2022 | Nomogram for Prediction of Postoperative Delirium after Deep Brain Stimulation of Subthalamic Nucleus in Parkinson's Disease under General Anesthesia | Outcome measure discrepancy:delirium |
|  | Schoffer, Kerrie L  Henderson, Robert D  O'Maley, Karen  O'Sullivan, John D | 2007 | Nonpharmacological treatment, fludrocortisone, and domperidone for orthostatic hypotension in Parkinson's disease | Study type mismatch: randomized controlled trial |
|  | Perez-Lloret, S  Negre-Pages, L  Ojero-Senard, A | 2012 | Oro-buccal symptoms (dysphagia, dysarthria, and sialorrhea) in patients with Parkinson's disease: preliminary analysis from the French COPARK cohort | Outcome measure discrepancy:Oral and cheek symptoms |
|  | Park, Jinse  Kim, Hee-Tae  Park, Kang Min  Ha, Sam Yeol | 2017 | Orthostatic dizziness in Parkinson's disease is attributed to cerebral hypoperfusion: A transcranial doppler study | Outcome measure discrepancy:dizziness |
|  | Sclater, A  Alagiakrishnan, K | 2004 | Orthostatic hypotension - A primary care primer for assessment and treatment | Study type mismatch: review |
|  | Freeman, Roy  Abuzinadah | 2018 | Orthostatic Hypotension <i>JACC</i> State-of-the-Art Review | Study type mismatch: review |
|  | Mussi, Chiara  Ungar, Andrea  Salvioli, Gianfranco | 2009 | Orthostatic Hypotension As Cause of Syncope in Patients Older Than 65 Years Admitted to Emergency Departments for Transient Loss of Consciousness | Study population mismatch：Patients over 65 years of age who have lost consciousness |
|  | Isik, Ahmet Turan  Dost, Fatma Sena | 2023 | Orthostatic hypotension in dementia with Lewy bodies: a meta-analysis of prospective studies | Study population mismatch：dementia patient |
|  | Jiang, Qirui  Zhang, Lingyu  Lin, Junyu | 2023 | Orthostatic Hypotension in Multiple System Atrophy: Related Factors and Disease Prognosis | Study population mismatch：Patients with multiple system atrophy |
|  | Mei, Shanshan  Wang, Xue | 2024 | Orthostatic Hypotension: a clinical marker for the body-first subtype of patients with Parkinson's Disease | Study type mismatch: Randomized controlled trial |
|  | Liang, Huey-Wen  Huang, Ya-Ping  Pan, Shin-Liang | 2015 | Parkinson disease and risk of acute myocardial infarction: A population-based, propensity score-matched, longitudinal follow-up study | Study population mismatch：Patients with acute myocarditis |
|  | Grosu, Laura  Grosu, Alin Ionut  Crisan, Dana | 2023 | Parkinson's disease and cardiovascular involvement: Edifying insights (Review) | Study type mismatch: Review |
|  | Pathak, Atul  Senard, Jean-Michel | 2004 | Pharmacology of orthostatic hypotension in Parkinson's disease: from pathophysiology to management. | Study type mismatch: Review |
|  | Fisher, A A  Davis, M W  Srikusalanukul, W | 2005 | Postprandial hypotension predicts all-cause mortality in older, low-level care residents | Study population mismatch：Community seniors aged 65 and above |
|  | Guo, Yu  Liu, Feng-Tao  Hou, Xiao-He | 2021 | Predictors of cognitive impairment in Parkinson's disease: a systematic review and meta-analysis of prospective cohort studies | Outcome measure discrepancy:cognitive impairment |
|  | Anang, Julius B M  Gagnon, Jean-Francois  Bertrand, Josie | 2014 | Predictors of dementia in Parkinson disease A prospective cohort study | Outcome measure discrepancy:dementia |
|  | Umehara, Tadashi  Nakahara, Atsuo  Matsuno, Hiromasa | 2016 | Predictors of postprandial hypotension in elderly patients with de novo Parkinson's disease | Outcome measure discrepancy:postprandial hypoglycemia |
|  | Enrique Asensio, L  Andrea Aguilera, C  de Los Angeles | 2011 | Prevalence of orthostatic hypotension in a series of elderly Mexican institutionalized patients | Study population mismatch：elderly |
|  | Schreglmann, S R  Buchele, F  Sommerauer, M | 2017 | Pyridostigmine bromide versus fludrocortisone in the treatment of orthostatic hypotension in Parkinson's disease - a randomized controlled trial | Study type mismatch: Randomized controlled trial |
|  | Schreglmann, S R  Buchele, F  Kaegi, G  Baumann, C R | 2018 | Pyridostigmine bromide versus fludrocortisone in the treatment of orthostatic hypotension in Parkinson's disease - reply | Study type mismatch: Randomized controlled trial |
|  | Biaggioni, Italo  Freeman, Roy  Mathias | 2015 | Randomized Withdrawal Study of Patients With Symptomatic Neurogenic Orthostatic Hypotension Responsive to Droxidopa | Study type mismatch: Randomized controlled trial |
|  | Biswas, Debashis  Karabin, Beverly  Turner, Debra | 2019 | Role of nurses and nurse practitioners in the recognition, diagnosis, and management of neurogenic orthostatic hypotension: a narrative review | Study type mismatch:review |
|  | Weiss, Avraham  Beloosesky | 2016 | The association between orthostatic hypertension and all-cause mortality in hospitalized elderly persons | Study population mismatch：hospitalized elderly people |
|  | Soysal, Pinar  Aydin, Ali Ekrem  Okudur, Saadet Koc | 2016 | When should orthostatic blood pressure changes be evaluated in elderly: 1st, 3rd or 5th minute? | Study population mismatch：elderly people |
|  | Nozaki, S  Kang, J  Miyai, I  Matsumura, T | 1993 | [Postprandial hypotension in Parkinson's disease--the incidence and risk factor]. | postprandial hypotension |
|  | Choi, Ja Young  Han, Kyungdo  Kim, Yong Wook | 2023 | Association between Low Blood Pressure and Subsequent Risk of Parkinson's Disease in Older Adults Aged ≥75 Years. | Study population mismatch：Elderly patients aged 75 years or older |
|  | Cavallini, A  Micieli, G  Martignoni, E  Blandini, F  Fariello, R  Nappi, G | 1991 | Cardiopressor effects of short-term treatment with cabergoline in L-dopa stable responder parkinsonian patients: relevance of postprandial hypotension. | Outcome measure discrepancy：postprandial hypotension |
|  | Mehagnoul-Schipper, D J  Boerman, R H  Hoefnagels, W H  Jansen, R W | 2001 | Effect of levodopa on orthostatic and postprandial hypotension in elderly Parkinsonian patients. | Study type mismatch: Randomized controlled trial |
|  | Gujjar, Arunodaya  Al-Mamari, Ali  Jacob, P C  Jain, Rajiv | 2010 | Extrapontine myelinolysis as presenting manifestation of adrenal failure: a case report. | Study type mismatch: case report |
|  | DiFrancisco-Donoghue, Joanne | 2019 | Nicotine Gum as a Therapeutic Approach for Low Blood Pressure in Parkinson's Disease: A Randomized Pilot Study. | Study type mismatch: Randomized controlled trial |
|  | "EUCTR SK" | 2013 | A study to assess the clinical benefit of Midodrine Hydrochloride in patients with symptomatic orthostatic hypotension | Study type mismatch: Randomized controlled trial |
|  | "EUCTR CZ" | 2013 | A study to assess the clinical benefit of Midodrine Hydrochloride in patients with symptomatic orthostatic hypotension | Study type mismatch: Randomized controlled trial |
|  | Guseva, O V  Zhukova, N G | 2021 | [The high blood pressure in patients with Parkinson's disease]. | Outcome measure discrepancy：hypertension |
|  | Shadmand, Mehdi  Elliott, Brian | 2024 | A retrospective analysis of neurogenic orthostatic hypotension in long-term care facility residents with recurrent falls. | Study population mismatch：Hospitalized patients who repeatedly fall |
|  | Rolbin, S H  Levinson, G | 1979 | Dopamine treatment of spinal hypotension decreases uterine blood flow in the pregnant ewe. | Study population mismatch：diabetic patient |
|  | Myers, M G  Kearns, P M  Kennedy, D S  Fisher, R H | 1978 | orthostatic hypotension and diuretic therapy in the elderly. | Study population mismatch： elderly |
|  | Robinson, L  Kimpinski, K | 2019 | Neurogenic orthostatic hypotension impairs information processing speed and attention | Study type mismatch: Randomized controlled trial |
|  | Chuang, C S  Wu, S L | 2019 | Prevalence of orthostatic hypotension and postprandial hypotension in neurodegenerative diseases with synucleinopathy: A systematic review and meta-analysis | Study type mismatch: meta-analysis |
|  | Negida, A  Attia, D | 2017 | Safety and efficacy of pardoprunox for patients with Parkinson's disease: A meta analysis | Study type mismatch: meta-analysis |
|  | DiFrancisco-Donoghue, J | 2016 | Use of nicotine gum to treat acute low blood pressure in Parkinson's disease | Study type mismatch: Randomized controlled trial |
|  | Hauser, R | 2015 | Integrated efficacy and safety analyses of droxidopa for symptomatic neurogenic orthostatic hypotension | Study type mismatch: Randomized controlled trial |
|  | Camargo, C | 2015 | Orthostatic hypotension and its relationship to the brain circulation of patients with parkinson's disease: A transcranial doppler assessment | Study type mismatch: case-control study |
|  | Lieberman, A | 2014 | An investigation of recurrent falls in parkinson disease (PD) | Outcome measure discrepancy：fall |
|  | Kaufmann, H | 2014 | Integrated safety of droxidopa for neurogenic orthostatic hypotension | Study type mismatch: Randomized controlled trial |
|  | Nakamura, T  Hirayama, M | 2014 | Role of cardiac sympathetic nerves in preventing orthostatic hypotension in Parkinson's disease | Study type mismatch: Randomized controlled trial |
|  | Umehara, T  Toyoda, C | 2014 | Postprandial hypotension in de novo Parkinson's disease: A comparison with orthostatic hypotension | Study type mismatch: Randomized controlled trial |
|  | Isaacson, S  Hauser, R | 2013 | Droxidopa Treatment Impact on Orthostatic Symptoms and Standing Systolic Blood Pressure in Patients with Parkinson's Disease (PD) and Symptomatic Neurogenic Orthostatic Hypotension (NOH) | Study type mismatch: Randomized controlled trial |
|  | Chitsaz, A | 2012 | Frequency distribution of orthostatic hypotension, nocturnal hypertension and postprandial hypotention in patients with Parkinson's disease: A case-control study | Study type mismatch：case-control study |
|  | Kaufmann, H  Mathias, C | 2009 | Treatment with droxidopa - A phase III multinational, placebo-controlled, parallel group, withdrawal-design study in subjects with neurogenic orthostatic hypotension and non-diabetic autonomic neuropathy | Study type mismatch: Randomized controlled trial |
|  | Krygowska-Wajs, A  Furgała, A | 2002 | Early diagnosis of orthostatic hypotension in idopathic Parkinson's disease | Study type mismatch: Randomized controlled trial |
|  | Siguroardóttir, G R  Nilsson, C | 2001 | Cardiovascular effects of domperidone in patients with Parkinson's disease treated with apomorphine | Study type mismatch: Randomized controlled trial |
|  | Durrieu, G  Senard, J M | 1990 | Blood pressure and plasma catecholamines in never-treated parkinsonian patients: effect of a selective D1 agonist (CY 208-243) | Study type mismatch: experimental research |
|  | Ishizaki, F  Harada, T | 1996 | Prolonged QTc intervals in Parkinson's disease relation to sudden death and autonomic dysfunction | Study type mismatch: experimental research |
|  | Loew, F  Gauthey, L | 1995 | Postprandial hypotension and orthostatic blood pressure responses in elderly Parkinson's disease patients | Study type mismatch: experimental research |
|  | Piha, S J  Rinne, J O | 1988 | Autonomic dysfunction in recent onset and advanced Parkinson's disease | Study type mismatch: experimental research |
|  | Kuroiwa, Y  Wada, T  Tohgi, H | 1987 | Measurement of blood pressure and heart-rate variation while resting supine and standing for the evaluation of autonomic dysfunction | Study type mismatch: experimental research |
|  | Li, M  Zhou, L  Ma, J | 2024 | Acupuncture at stellate ganglion combined with western medication for orthostatic hypotension in Parkinson's disease: a randomized controlled trial | Study type mismatch: experimental research |
|  | Cucinotta, F  Swinnen, B | 2024 | Short term cardiovascular symptoms improvement after deep brain stimulation in patients with Parkinson's disease: a systematic review | Study type mismatch: systematic review |
|  | Bane, A  Wilson, L  Jumper, J | 2024 | Effects of Blood Flow Restriction Resistance Training on Autonomic and Endothelial Function in Persons with Parkinson's Disease | Outcome measure discrepancy：Hemorrhoids |
|  | Giannini, G  Minardi, R  Barletta, G | 2024 | The Degree of Cardiovascular Autonomic Dysfunction is not Different in GBA-Related and Idiopathic Parkinson's Disease Patients: A Case-Control Instrumental Evaluation | Study type mismatch: case-control study |
|  | Hu, J  Chi, J  Cai, H | 2024 | Effect of orthostatic hypotension on long-term prognosis of elderly patients with stable coronary artery disease: a retrospective cohort study | Study population mismatch：Elderly patients with stable coronary heart disease |
|  | Khalil, I  Sayad, R | 2024 | Cardiovascular dysautonomia and cognitive impairment in Parkinson's disease (Review) | Study type mismatch: Review |
|  | de Aquino, C H  Moscovich, M | 2024 | Fundamentals of deep brain stimulation for Parkinson’s disease in clinical practice: part 1 | Study type mismatch: Review |
|  | Yang, R  Cai, J | 2023 | Orthostatic Blood Pressure Changes and Cognitive Function: Insights From the SPRINT MIND Trial | Study population mismatch：Cognitive impairment patient |
|  | Prajjwal, P  Flores Sanga, H S  Acharya, K | 2023 | Parkinson's disease updates: Addressing the pathophysiology, risk factors, genetics, diagnosis, along with the medical and surgical treatment | Study type mismatch: Review |
|  | Poston, K L  Thaler, A | 2022 | Diagnosis and Medical Management of Parkinson Disease | Study type mismatch: Review |
|  | Lamotte, G  Lenka, A | 2022 | Orthostatic Hypotension in Parkinson Disease: What Is New? | Study type mismatch: Review |
|  | Zhou, W  Sinn, D I  Jaradeh, S | 2022 | High Resolution Anorectal Manometry Findings in Men and Women With Parkinson's Disease, Using London Classification | Outcome measure discrepancy：Anal and rectal diseases |
|  | Ananthavarathan, P  Patel, B | 2022 | A systematic review of non-motor symptoms in atypical parkinsonian syndromes | Study type mismatch: Review |
|  | Alves, M  Pita Lobo, P | 2022 | Neuroimaging cerebrovascular biomarkers in Parkinson’s disease | Study type mismatch: case-control study |
|  | Torino, C  Tripepi, R | 2021 | Clinical epidemiology of systolic and diastolic orthostatic hypotension in patients on peritoneal dialysis | Study population mismatch：Peritoneal dialysis patients |
|  | McDonagh, S T J | 2021 | Prevalence of orthostatic hypotension in primary, community and institutional care: a systematic review and meta-analysis | Study type mismatch: Review |
|  | Farrell, M C  Shibao, C A | 2020 | Morbidity and mortality in orthostatic hypotension | Study type mismatch: Review |
|  | Sutantoyo, F F  Sugianto, P | 2020 | The Diagnostic Challenges in Patient with Multiple System Atrophy: A Case Report | Study type mismatch: case report |
|  | De Jager, L  Vidigal, C B | 2020 | Participation of the iNOS isoform in cardiovascular and autonomic dysfunction in male rats submitted to parkinsonism by 6-OHDA | Study type mismatch: Randomized controlled trial |
|  | Cutsforth-Gregory, J K | 2019 | Neurogenic Orthostatic Hypotension in Parkinson Disease: A Primer | Study type mismatch: Review |
|  | Wang, A S  Gunzler, S A | 2019 | Systematic review of the pharmacoeconomics of Parkinson disease medications | Study type mismatch: mete analysis |
|  | Roberson, K B  Signorile, J F | 2019 | Hemodynamic responses to an exercise stress test in Parkinson's disease patients without orthostatic hypotension | Study type mismatch: Randomized controlled trial |
|  | Matar, E | 2019 | HOW TO DIAGNOSE LEWY BODY DEMENTIA? PREVALENCE AND UNDERLYING RELATIONSHIP BETWEEN CLINICAL AND NEUROPSYCHOLOGICAL FEATURES OF DLB | Study type mismatch: randomized controlled trial |
|  | McCullough, P A | 2019 | Treatment of Orthostatic Hypotension Due to Autonomic Dysfunction (Neurogenic Orthostatic Hypotension) in a Patient with Cardiovascular Disease and Parkinson's Disease | Study type mismatch: case report |
|  | Panuccio, V A  Tripepi, R | 2019 | Clinical epidemiology of systolic and diastolic orthostatic hypotension in patients on peritoneal dialysis | Study population mismatch：Peritoneal dialysis patients |
|  | Liu, W  Liu, W | 2019 | Clinical study of twenty-four hour non-invasive ambulatory blood pressure and heart rate monitoring in hospitalized patients with Parkinson's disease | Study type mismatch: Randomized controlled trial |
|  | Kymes, S  François, C  Sullivan, C | 2019 | Effects of droxidopa treatment for neurogenic orthostatic hypotension in patients concomitantly on dopa decarboxylase inhibitors | Study type mismatch: Randomized controlled trial |
|  | Seppi, K  Ray Chaudhuri, K  Coelho, M | 2019 | Update on treatments for nonmotor symptoms of Parkinson's disease—an evidence-based medicine review | Study type mismatch: Review |
|  | Duan, X  Zhang, L | 2018 | FXTAS-like Phenotype with p.P626L Missense Mutation: A case report | Study type mismatch: case report |
|  | Ellenbogen, A  Hauser, R  Isaacson, S | 2018 | Inhaled levodopa administered with oral carbidopa/levodopa for early morning off symptoms in patients with Parkinson's disease: Safety assessment | Study type mismatch: Randomized controlled trial |
|  | Ray Chaudhuri, K  Sauerbier, A  Ferreira, J | 2018 | Dopaminergic adverse-events in COMTnaïve patients starting adjunctive therapy with opicapone: The BIPARK-I doubleblind experience | Study type mismatch: Randomized controlled trial |
|  | Palma, J A  Kaufmann, H | 2017 | Epidemiology, Diagnosis, and Management of Neurogenic Orthostatic Hypotension | Study type mismatch: Review |
|  | Lewis, S J  Gangadharan, S | 2016 | Parkinson's disease in the older patient | Outcome measure discrepancy：fall |
|  | McDonald, C  Newton, J L | 2016 | Orthostatic hypotension and cognitive impairment in Parkinson's disease: Causation or association? | Study type mismatch: Review |
|  | Rosso, M  Merola, A  Espay, A J | 2016 | Parkinson's disease prodromal dysautonomia versus pure autonomic failure and MSA | Study type mismatch: case report |
|  | Stankard, A  Walsh, T | 2016 | Orthostatic hypotension and efforts to manage a difficult case | Study type mismatch: case report |
|  | Heiry, M  Cortez, M | 2016 | Autonomic dysfunction in dementia with lewy bodies, a case series | Study type mismatch: case report |
|  | El-Saifi, N  Moyle, W  Jones, C  Tuffaha, H | 2016 | Quetiapine safety in older adults: A systematic literature review | Study type mismatch: Review |
|  | Liepelt-Scarfone, I  Pilotto, A  Müller, K | 2015 | Autonomic dysfunction in subjects at high risk for Parkinson’s disease | Study type mismatch: Randomized controlled trial |
|  | Jones, P K  Shaw, B H | 2015 | Orthostatic hypotension: Managing a difficult problem | Study type mismatch: Review |
|  | Fanciulli, A | 2015 | Cardiovascular autonomic failure in α-Synucleinopathies: Epidemiological aspects | Study type mismatch: Review |
|  | Lotfinia, M | 2015 | Clinical course of migraine in parkinson patients | Outcome measure discrepancy：migraine |
|  | Schrag, A  Horsfall, L  Walters, K | 2015 | Prediagnostic presentations of Parkinson's disease in primary care: A case-control study | Study type mismatch: case-control study |
|  | Kanjwal, K  George, A | 2015 | Orthostatic hypotension: Definition, diagnosis and management | Study type mismatch: Review |
|  | Hauser, R A  Isaacson, S | 2015 | Droxidopa for the Short-Term Treatment of Symptomatic Neurogenic Orthostatic Hypotension in Parkinson's Disease (nOH306B) | Study type mismatch: randomized controlled trial |
|  | Slevin, J T  Fernandez, H H  Zadikoff, C | 2015 | Long-term safety and maintenance of efficacy of levodopa-carbidopa intestinal gel: An open-label extension of the double-blind pivotal study in advanced Parkinson's disease patients | Study type mismatch: Randomized controlled trial |
|  | Sandoval-Rincón, M  Sáenz-Farret, M  Miguel-Puga, A | 2015 | Rational pharmacological approaches for cognitive dysfunction and depression in Parkinson's disease | Study type mismatch: Review |
|  | Akpinar, C K  Aygun, D  Yilmaz, O  Onar, M K | 2015 | Electrocardiographic changes in the off-medication state in Parkinson's disease | Outcome measure discrepancy：ECG changes |
|  | Li Lixia  Lian Tenghong | 2020 | Characteristics of orthostatic hypotension in patients with Parkinson's disease and its impact on cognitive function | Language mismatch |
|  | Xiao Renqing  Song Lu  Zhao Jiahao | 2023 | Preliminary analysis of the clinical characteristics of circadian rhythm disorders in blood pressure in patients with Parkinson's disease and their impact on orthostatic hypotension | Language mismatch |
|  | Li Lingxiao  Zhou Fubo | 2024 | A Study on the Similarities and Differences in Orthostatic Hypotension Between Patients with Parkinson's Disease and Multiple System Atrophy | Language mismatch |
